# scMSI: Accurately inferring the sub-clonal Micro-Satellite status by an integrated deconvolution model on length spectrum

**DOI:** 10.1371/journal.pcbi.1012608

**Published:** 2024-12-02

**Authors:** Yuqian Liu, Yan Chen, Huanwen Wu, Xuanping Zhang, Yuqi Wang, Xin Yi, Zhiyong Liang, Jiayin Wang

**Affiliations:** 1 School of Computer Science and Technology, Xi’an Jiaotong University, Xi’an, China; 2 Department of Pathology, State Key Laboratory of Complex Severe and Rare Disease, Molecular Pathology Research Center, Peking Union Medical College Hospital, Chinese Academy of Medical Sciences and Peking Union Medical College, Beijing, China; 3 Geneplus Beijing Institute, Beijing, China; Children’s National Hospital, George Washington University, UNITED STATES OF AMERICA

## Abstract

Microsatellite instability (MSI) is an important genomic biomarker for cancer diagnosis and treatment, and sequencing-based approaches are often applied to identify MSI because of its fastness and efficiency. These approaches, however, may fail to identify MSI on one or more sub-clones for certain cancers with a high degree of heterogeneity, leading to erroneous diagnoses and unsuitable treatments. Besides, the computational cost of identifying sub-clonal MSI can be exponentially increased when multiple sub-clones with different length distributions share MSI status. Herein, this paper proposes “scMSI”, an accurate and efficient estimation of sub-clonal MSI to identify the microsatellite status. scMSI is an integrative Bayesian method to deconvolute the mixed-length distribution of sub-clones by a novel alternating iterative optimization procedure based on a subtle generative model. During the process of deconvolution, the optimized division of each sub-clone is attained by a heuristic algorithm, aligning with clone proportions that adhere optimally to the sample’s clonal structure. To evaluate the performance, 16 patients diagnosed with endometrial cancer, exhibiting positive responses to the treatment despite having negative MSI status based on sequencing-based approaches, were considered. Excitingly, scMSI reported MSI on sub-clones successfully, and the findings matched the conclusions on immunohistochemistry. In addition, testing results on a series of experiments with simulation datasets concerning a variety of impact factors demonstrated the effectiveness and superiority of scMSI in detecting MSI on sub-clones over existing approaches. scMSI provides a new way of detecting MSI for cancers with a high degree of heterogeneity.

## Author summary

Microsatellites are short, repetitive sequences of DNA, and their instability (MSI) is an important marker for cancer diagnosis and treatment. However, tumors often consist of diverse groups of cells, or sub-clones, and existing sequencing methods often fail to detect MSI that occurs only in some sub-clones. This can lead to incorrect diagnoses and prevent patients from receiving the most effective therapies. To solve this problem, we developed a new computational method named as scMSI to accurately identify MSI of sub-clones within a tumor. scMSI utilizes advanced statistical techniques to deconvolute the complex mixture of genetic mutations. As a result, we can use scMSI to detect sub-clonal MSI that other methods might miss. In the testing, we examined scMSI on samples from 16 patients with endometrial cancer, who had been incorrectly labeled as MSI-negative by existing methods. Our method successfully identified MSI in sub-clones, showing that scMSI outperforms existing tools. Additionally, simulation experiments under various conditions further confirmed the effectiveness of scMSI in detecting sub-clonal MSI. By improving the detection of MSI in cancers with a high degree of heterogeneity, scMSI can enhance cancer diagnosis and treatments more effectively.

## 1. Introduction

**Fig 1:**
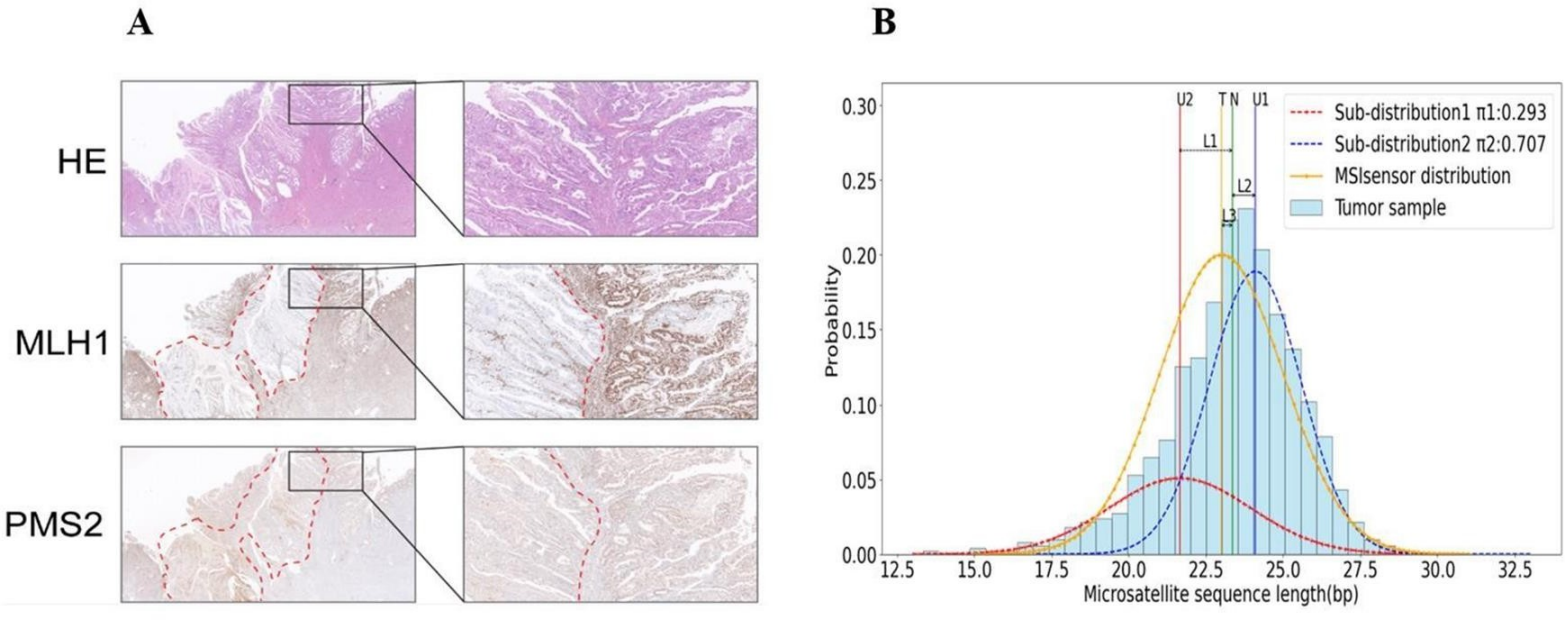
An endometrial cancer patient with tumor-normal paired samples. (A) The immunohistochemical result show subclonal immunohistochemical loss of MMR expression in a variable proportion of tumor cells in tumor sample. (B) MS length distributions in paired samples. T, N represents the mean of MS length distribution in tumor sample and normal sample, respectively. U1 and U2 respectively represent the mean value of MS distribution in each clone. MSIsensor distribution represents the MS length distribution curve fitted based on the statistical test assumption of MSIsensor.

Microsatellites (MS) denote repetitive DNA sequences consisting of short repeats varying from 1–6 dp. Their instability indicates the abnormalities in the number of MS repetitions, arising from defects in the DNA mismatch repair (MMR) system, which is responsible for correcting DNA replication errors [1]. As a matter of course, MS instability (MSI) stands out as a crucial genomic biomarker in various cancers [2–9], and holds significant therapeutic implications for both cancer diagnosis and treatment [10–12]. MSI-related cancers often present a high degree of heterogeneity. Our recent study revealed that some patients exhibit evident sub-clonal immunohistochemical loss of MMR expression, as shown by a sample from an endometrial cancer patient in **Fig 1** [13]. We categorized this patient as partially MSI-positive. However, existing sequencing-based methods lack the ability to accurately detect such partial MSI-positives and often misclassify them as MSI-negative, resulting in false-negative detection errors that deprive patients of potential treatment benefits. Meanwhile, it is important to mention that even when reported as the positive cases, it has been advised that treatment and monitoring for patients with partial MSI-positive should differ from those with complete MSI-positive [13,14]. Hence, there is an unmet important clinical need to develop a sub-clone MSI calling algorithm that can aid in decision-making for MSI-stratified therapy [15].

Why are existing methods unable to accurately detect the microsatellite event on different sub-clones? These approaches capture the features from sequencing data to detect MSI events, which primarily differ in their focus on either length distribution or mutation burden. The length distribution strategy counts the number of sequencing reads carrying microsatellites of different lengths and determines MSI events by statistical methods, such as MSIsensor, MSIsensor-pro, mSINGS and MANTIS [16–19]. The mutation burden strategy, such as MSIsensor-ct, MSIpred and MIRMMR [20–22], collects mutations around some microsatellite areas, and pre-trained machine learning models estimate MSI status. However, these approaches have a common problem, that is, they all ignore tumor clonality in real clinical scenarios. When they calculate the length distribution, it implies a strong hypothesis that the lengths of one microsatellite in all clones follow the same unimodal distribution; while for mutation burden strategies, purity, variant allelic frequency or sub-clonal proportions are never incorporated into the model via either the input or the structure or components of the pre-trained models. Revisiting the partial MSI-positive case in **Fig 1**, **Fig 1B** presents the length distribution at the corresponding microsatellite area after we sequenced it. According to the histogram, it clearly should not be a single unimodal distribution. When the histogram is fitted by the curve of a normal distribution (single unimodal distribution assumption by MSIsensor), it exposes a significant bias from the multiple sub-clonal curves (sub-length distributions). As a result, this partial MSI-positive patient is reported as MSI-negative. Thus, it is Achilles’s heel for the existing approaches to suppose a consistent clonality, while the observed length distributions or mutations are a mixture of sub-clones.

To solve this issue, could we simply use multimodal distributions? It seems not easy, not only due to the high degree of heterogeneity (a large number of sub-clones), e.g., endometrial cancers, but also because sub-clones may share the same microsatellite events during clonal expansion. In the case of high-dimensional multivariable analysis, the deconvolution on an observed length distribution may fall into a calculation situation where the posterior probability is not feasible or has no analytical solution for a solved integral with limited prior information. In addition, the combinations of sub-clonal microsatellite events are controlled by the same clonal structure across the whole sample. It is another crucial issue in computation to utilize the global clonal structure to guide local inference. Therefore, the potential computational method has to consider these two issues simultaneously and obtain the globally optimal parameters and states by deconvolution.

Herein, we present scMSI, which is an integrative Bayesian method based on a subtle generative model and a novel alternating iterative optimization procedure, to identify sub-clonal MSI events on tumor-normal paired sequencing data. It infers the posterior distribution of each sub-clone length expression from observed data using the clonal structure information as the prior. A matrix-wise generative model with clone fractions and corresponding length expressions describes the length distribution of sub-clones. An additional binary relationship matrix is introduced into the model as part of the optimization parameters to help us discretize the length distribution that may potentially be shared by several sub-clones. To avoid the complex multidimensional integration of the edge likelihood function in the high-dimensional multivariable environment, a cluster of tractable distributions is employed to approximate the true distribution during the inference. Hence, a feasible solution is delivered with reduced computational complexity. In addition, scMSI is able to accelerate the estimation process by inferring the status of multiple loci in parallel.

To evaluate the performance, 16 patients diagnosed with endometrial cancer, exhibiting positive responses to the treatment despite having negative MSI status based on sequencing-based approaches, were collected. scMSI reported MSI on sub-clones successfully, and the findings matched the conclusions on immunohistochemistry. We compare our algorithm with MSIsensor, MSIsensor-pro, mSINGS and MANTIS on real datasets. In addition, testing results on a series of experiments with simulation datasets concerning a variety of impact factors demonstrated the effectiveness and superiority of scMSI in detecting MSI on sub-clones over existing approaches. scMSI provides a new way of detecting MSI for cancers with a high degree of heterogeneity.

## 2. Materials and method

### 2.1. Materials

#### 2.1.1. Ethics statement

This study was conducted according to the principles expressed in the Declaration of Helsinki. Ethical approval for this study was granted by the Institutional Review Board of Peking Union Medical College Hospital (No. S-K2006). Written informed consent was obtained from all subjects.

#### 2.1.2. Patient cohort collection

In this research, we recruited 16 endometrial cancer patients from Peking Union Medical College Hospital, and surgical tumors were collected from each patient, which are formalin-fixed and paraffin-embedded (FFPE). For 16 endometrial cancer patients, FFPE tissues from all cases were immunohistochemically stained for MMR proteins MLH1, MSH2, MSH6, and PMS2 using the Envision Plus detection system (Dako, Carpinteria, CA). Clonal/Sub-clonal loss of MMR expression was defined as abrupt and complete regional loss of expression of any MMR protein as previously reported [13]. Intervening stromal positivity in the regions of absent tumor cell staining served as internal control to exclude heterogeneous immunohistochemical staining due to suboptimal preanalytic handling. The immunostaining results of MMR protein in these cases were evaluated by two experienced pathologists (H.W and Z.L). The collected cohort of 16 endometrial cancer patients were all MSI samples.

For DNA and library preparation, DNA was independently extracted from microdissected FFPE samples using the Promega Maxwell RSC DNA FFPE kit (Promega, Madison, WI, USA) at Peking Union Medical College Hospital and concentrations were measured using a Qubit fluorometer and the Qubit dsDNA HS (High Sensitivity) Assay Kit (Invitrogen, Carlsbad, CA, USA). Fragment DNA was then added to Illumina index adapters for library construction using the KAPA Library Preparation Kit (Kapa Biosystems, Wilmington, MA, USA) and sequenced on a HiSeq3000 sequencing system.

We mapped the sequencing data to human reference genome (hg19) via BWA [23]. The microsatellite loci consist of single nucleotide repeats are scanned as they are reported the most sensitive and specific for MSI status. We combined the scanned sites used by colony core [24] with PCR detection sites NR-21, BAT-26, NR-27, BAT-25, NR-24, NR-22 and Mono-27 to determine 15 microsatellite sites, which can be found in **Table H in S1 Text**. In the meantime, we used MSIsensor to establish the length distributions for comparison. For each locus of marked loci, all read pairs with at least one mapped read within 2 kb of the locus were extracted from the paired normal tumor alignment files. A dictionary is then created using concatenating the flanking sequences to all possible repeat lengths, where repeat lengths range from 0 to read length minus 10. Finally, the number of instances corresponding to a set of sequencing reads at the MS locus is counted, and the frequency distribution of the reads is obtained, thereby reconstructing the allele distribution. The obtained allele distribution is transformed to obtain the final microsatellite length data. In addition, we used sciclone [25] to obtain the somatic mutations and the numbers and proportions of sub-clones.

#### 2.1.3. Sequencing data for simulation experiments

Human reference genome (hg19) is selected as the reference genome. We designed a series of configurations with various repeat lengths, repeat units and breakpoints of microsatellite areas. We used GSDcreator [26] to implant the designed microsatellite to selected sites in the form of a mixture of Gaussian distributions. The number of microsatellites corresponding to the respective repeats of each locus was obtained by multiplying the mutation frequency of the microsatellites corresponding to the repeats by the sequencing depth. When all microsatellites are implanted, we merge all read files, and the mapping and variant calling process was the same as we adopted in section 2.1.1.

### 2.2. The model and algorithm

**Fig 2:**
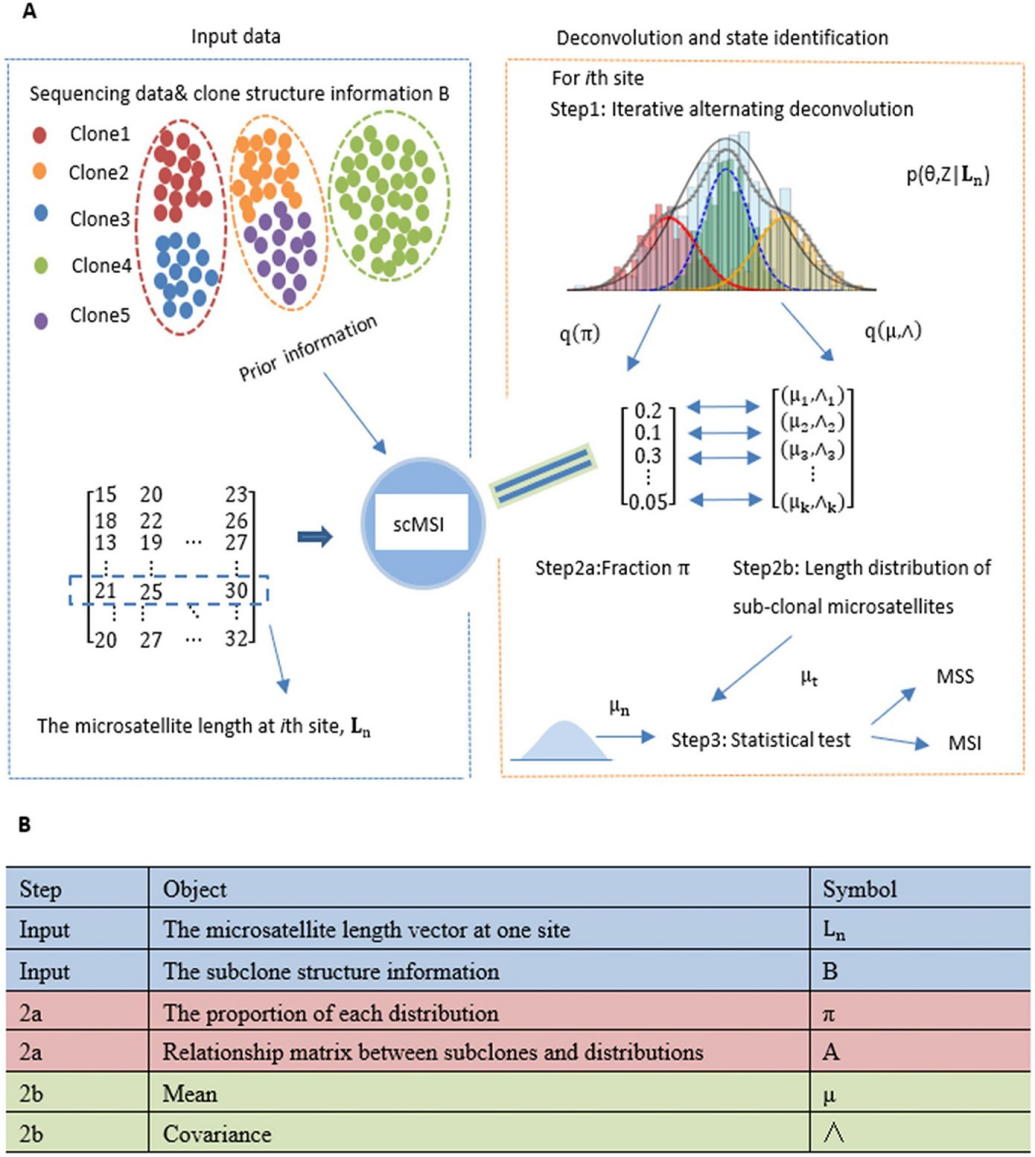
The flowchart of scMSI. (A) Algorithmist flow of scMSI. (B) Variables shown in (A).

scMSI is comprised of two functional modules: (1) a module that infers the fraction and length distribution of each sub-clone for one sample. (2) a module designed to determine the microsatellite status of each sub-clone by a statistical test on length distribution via deconvolution. The algorithm flow of scMSI and the relevant variables are shown in **Fig 2A and 2B**.

#### 2.2.1. Model specification

A sequencing sample contains multiple microsatellites loci. The relationship between the length distribution of each locus and the length distribution of microsatellites in each clone is defined as **MB** = **U**, where **M** represents the length of microsatellites at each marked locus in each clone, **B** represents the structural information of sub-clones, and **U** represents the actual observed length distribution matrix for the batch of microsatellite loci.

For each tagged microsatellite locus i∈[I] and each clone g∈[G−], h_i,g_ describes the length distribution of microsatellites in clone g at marker locus i.

$$T_{i}=\sum_{g=1}^{G}B_{g}h_{i,g}$$ (1)

where $B={\left(B_{g}\right)}_{g\in [G]}\in \Delta^{G-1}$ contains the mixed proportion for each sub-clone. T_i_ represents the microsatellite length distribution at the *i*th site, which is:

$$\underset{M}{\underset{︸}{\left[\begin{array}{ccc} f(h_{1,1}) & \ldots & f(h_{1,g})\\ f(h_{2,1}) & \vdots & f(h_{2,g})\\ \vdots & \vdots & \vdots \\ f(h_{i,1}) & \ldots & f(h_{i,g}) \end{array}\right]}}.\underset{B}{\underset{︸}{\left[\begin{array}{c} B_{1}\\ \vdots \\ B_{g} \end{array}\right]}}=\underset{U}{\underset{︸}{\left[\begin{array}{c} f(T_{1})\\ \vdots \\ f(T_{i}) \end{array}\right]}}$$ (2)

The length distribution for each sub-clone needs to be estimated. Here, the length distribution T_i_ of the *i*th microsatellite site in Eq (2) is a mixture distribution. We have two observations: 1) the real weights are completely regulated by the clone structure information. 2) the length distribution is approximately a normal distribution according to literatures [16–22,27]. We suppose that the distribution of each sub-clone is independent to each other. When the microsatellite lengths of clone G in the tumor obey the K normal distributions (K≤G), the lengths of clonal microsatellites in tumors follow a mixture of Gaussian distributions. Hence, scMSI models the microsatellite length of each clone using a multinomial distribution. The goal is to approximate the clonal length distribution for each microsatellite site across a sample with N bulk length data from sequencing reads as a linear combination of a small number of K bases, with K≪N. The mixed Gaussian model for one microsatellite site is defined as follows,

$$p(L)=\sum_{k=1}^{k}p(z_{k})p(L|z_{k})=\sum_{k=1}^{k}\pi_{k}N(L|\mu_{k},\wedge_{k}^{-1})$$ (3)

The length of the microsatellite from each read is recorded as **L**_n_(n = 1,……,N), and each **L**_n_ corresponds to a K-dimensional binary latent variable z_nk_, k = 1,…,K, where z_nk_ = 1 means that **L**_n_ belongs to the k-th cluster and satisfies $\sum_{k=1}^{K}z_{nk}=1$. Its marginal probability p(z_nk_ = 1) is given by the mixing coefficient π_k_, i.e., p(z_nk_ = 1) = π_k_.

Given the latent variables and component parameters, the conditional probability distribution of the observed length **L** can be obtained as

$$p(L|Z,\mu ,\wedge )=\prod_{n=1}^{N}\prod_{k=1}^{K}{N(L_{n}|\mu_{k},\wedge_{k}^{-1})}^{z_{nk}}$$ (4)

For parameter estimation of a mixed distribution, it is impossible to calculate the posterior probability distribution or the expectation of the posterior probability distribution in practical applications. This could be because the dimensions of the latent space are too high to directly estimate the posterior, or the form of the posterior probability distribution is too complicated to obtain an analytical solution of the expectation. Therefore, we adopt an approximation idea, the core of which is how to estimate the difficult-to-calculate probability density. Utilizing the idea of Bayesian inference in statistics, we regard all inferences about unknowns as the posteriors to be computed. Latent variables Z assist in controlling the data distribution and are extracted from the prior density p(Z); they are then linked to observations **L** by the likelihood p(**L**|Z).

**Fig 3:**
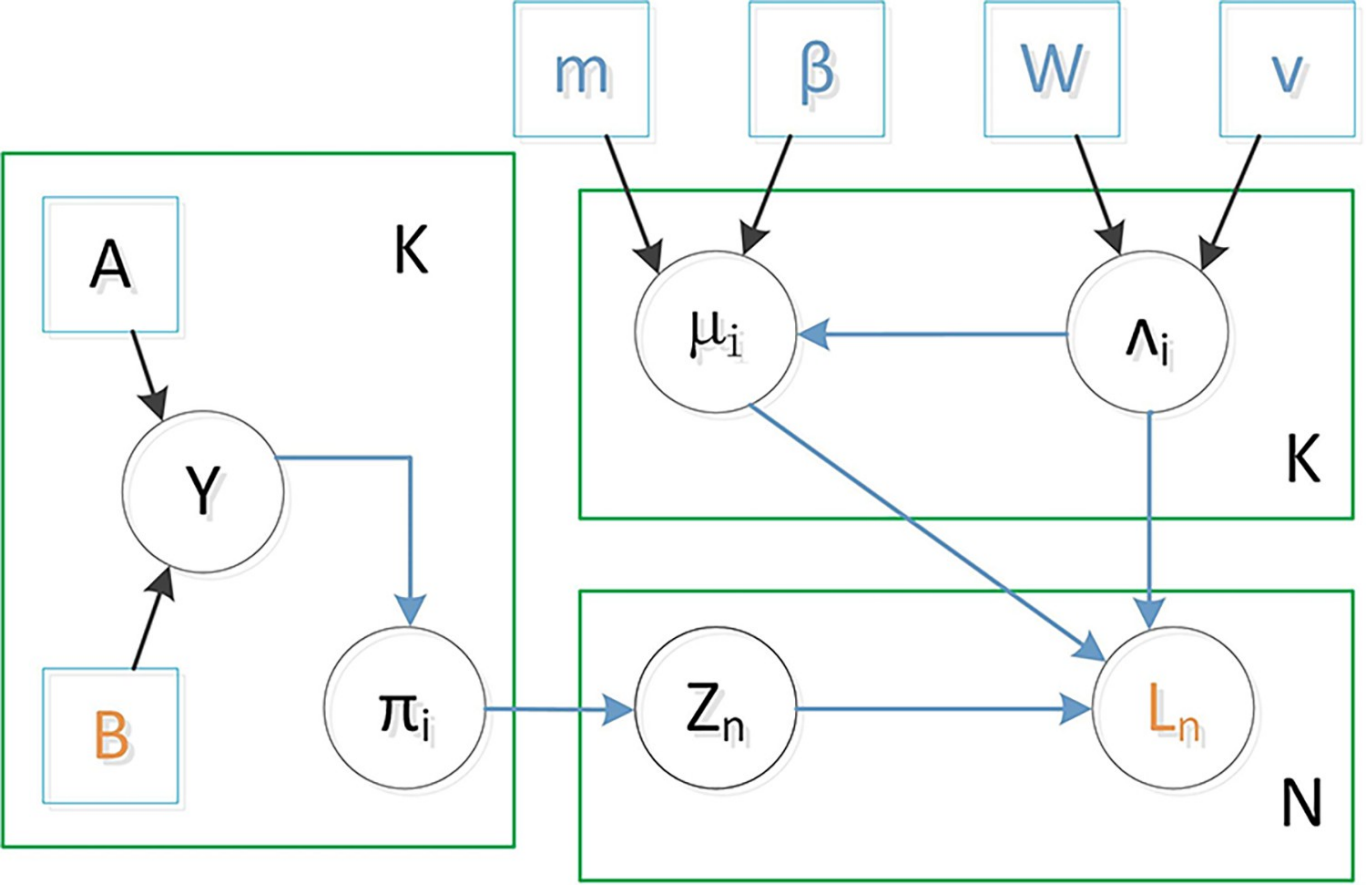
Probability graphical model of scMSI. $\mu ={\left\{\mu_{i}\right\}}_{i=1}^{K},\wedge ={\left\{\wedge_{i}\right\}}_{i=1}^{K}$ are the mean matrix and precision matrix related to the input variable L. A is a matrix, and each element **A**_**gk**_ in A is a binary vector of "1 of K". And **A**_**gk**_ **= 1** means that the length distribution of MS in the g-th clone belongs to the k-th cluster in the mixed model. **β, m** are the parameters of the conjugate prior distribution related to **μ**, and **w, v** are the parameters of the conjugate prior distribution related to ∧. Blue text marks hyper-parameters; orange marks observed variables; black marks latent variables.

The probabilistic graphical model that illustrates the statistical dependencies and the generative process for the observed mixed length is shown in **Fig 3**:

#### 2.2.2. Deconvolution on observed length distribution

A dual optimization objective is designed to approximate the conditional density of latent variables given the observed variables. We first assume a set of approximate density families Q, and define the relationship matrix A between each clone and each sub-distribution. Then, the dual goals of minimizing the difference between the fitted distribution and the true distribution and the Kullback-Leibler (KL) divergence are simultaneously achieved through coordinated stepwise optimization, that is, alternate iterative optimization of each member of the Q family and the relation matrix A.

a. Objective functions

In the case of considering the prior information of the parameters, we use a more manageable distribution q(θ, Z) to approximate the true posterior distribution of the parameters p(θ, Z|**L**). The log-likelihood function of the Bayesian model can be equivalently expressed as

$$\log\;p(L)=\log\;p(L,Z)-\log\;p(Z|L)=\log\frac{p(L,Z)}{p(Z|L)}$$ (5)

Further set the distribution q(z) as a variable term, then

$$\begin{array}{c} \log\frac{p(L,Z)}{p(Z|L)}=log\int q(Z)\frac{p(L,Z)q(Z)}{p(Z|L)q(Z)}dz\\ \geq \int q(Z)\{log\frac{p(L,Z)}{q(Z)}-log\frac{p(Z|L)}{q(Z)}\}dz\\ =EBLO(q(Z,\theta ))+KL(q||p)\\ \geq EBLO(q(Z,\theta )) \end{array}$$ (6)

The first inequality is introduced by Jensen’s inequality, and the second inequality is due to the fact that the KL divergence should be non-negative, and only when the approximation is equal to the true conditional posterior, the KL divergence is zero. Our goal is to make q(θ, Z)≈p(θ, Z|**L**), when q(θ, Z) and p(θ, Z|**L**) are equally distributed, the KL divergence is zero. Our ultimate goal is to minimize KL divergence is equivalent to maximize the model lower bound EBLO. Owing to the length distribution of microsatellites in tumors is a mixed distribution, the weights of sub-distributions randomly drawn from this distribution are controlled by the clonal structural information. Hence, we designed a quadratic integer programming objective function to discretize the mixing ratio of each distribution,

$$\begin{array}{c} \underset{A_{\mathrm{gk}}}{\min}\sum_{n=1}^{N}{\left\{\left[\sum_{k=1}^{K}\sum_{g=1}^{G}B_{g}A_{gk}N(L_{n}|\mu_{k},\sigma_{k})]-f(L_{n}\right)\right\}}^{2}\\ s.t.A_{gk}\in \{0,1\}\\ \sum_{k=1}^{K}A_{gk}=1 \end{array}$$ (7)

When the dual targets are satisfied, the length distribution parameters and the state of each cloned microsatellite can be obtained.

b. Inferring sub-clonal microsatellite length distribution

scMSI iteratively optimizes and updates each member of the Q family and the relationship matrix A under the constraint of dual optimization objectives. In the first stage, the mean-field theory is mainly used to estimate q(Z), the members of the Q family. In the second stage, the relationship matrix A is optimized under the control of the clone structure information through quadratic integer programming, ensuring that the local inference is consistent with the global clonal structure. Here, we employ the Gurobi solver [28]. These two optimizations are coordinated and gradually alternated to deconvolute the mixed microsatellite length distribution observed in the tumor sample. Below is a more detailed description.

We denote the joint probability distribution of parameters and samples as

p(L,Z,π,μ,∧)=p(L|Z,μ,∧)p(Z|π)p(π)P(μ|∧)P(∧) (8)

To obtain a tractable solution, some approximations are made to the true posterior distribution of the parameters. We assume that the posterior distribution of latent variables and model parameters can be decomposed between latent variables and parameters, i.e., q(Z,π,μ,∧)=q(Z)q(π,μ,∧).

The functional forms of the factors q(Z) and q(π, μ, ∧) will be automatically determined in the optimization process of the approximate distribution. The interdependence among the factors produces a circular optimization scheme. where the factors are properly initialized, and each factor is then iteratively updated by keeping the rest unchanged.

The analytical formula of the factor can be obtained by the mean field theory as

$$q_{j}^{*}(z_{j})=\frac{\exp\left(E_{i\neq j}[Inp(L,Z)]\right)}{\int exp(E_{i\neq j}[Inp(L,Z)])dz_{j}}$$ (9)

In general, for the solution of the entire model, the strategy we take is to iteratively and alternately update the factors q(Z) and q(π, μ, ∧) until the lower bound of the model is the largest. More specifically, it iteratively and alternately updates the approximate distribution of latent variables and model parameters π, μ, ∧. The process of solving and analyzing the whole model is shown in **Appendix A in S1 Text**. Let Δ = {z, π, μ, ∧} and P denotes the solution obtained from Eq (7).

The overall algorithm flow is shown in **Algorithm 1**. For detailed convergence analysis, please refer to [29–30].

**Algorithm 1**. Sub-Clonal Microsatellite Status Detection

**Input**: The allele distribution of each microsatellite in the normal-tumor paired sample, the number of clones M and the proportion

**Output**: the status of microsatellites in tumor subclone.

Begin:

Initialization

1: Initialize the number of components (k< = M), and initialize the model parameters

2: Initialize r_nk_ using k-means algorithm

while ${EBLO(q(\Delta ))}_{j+1}-{EBLO(q(\Delta ))}_{j}>\sigma \;do$

 $r_{nk}\leftarrow \frac{\rho_{nk}}{\sum_{i=1}^{K}\rho_{ni}}$

 A_gk_←P

 $Y_{k}\leftarrow B_{g}A_{gk}\sum_{k=1}^{K}Y_{k}$

 $m_{k}\leftarrow \frac{1}{\beta_{k}}(\beta_{0}m_{0}+N_{k}x_{k})$

 β_k_←N_k_+β_0_

 $w_{k}^{-1}\leftarrow w_{0}^{-1}+N_{k}S_{k}+\frac{{\beta_{0}N}_{k}}{\beta_{k}}{\left(x_{k}-m_{0})(x_{k}-m_{0}\right)}^{T}$

 v_k_←N_k_+v_0_

End

Statistical testing to determine the status of microsatellites in tumor subclone

Output:Microsatellite status in tumor subclone

## 3. Results and discussion

To evaluate performance and test characteristics of scMSI, experiments have been conducted on both the simulation dataset and the real cohort dataset.

For simulation study, scMSI is applied on the simulated dataset with a series of different configurations by varying clonal structure, sequencing depth and MS length distribution setting. The performance is evaluated using five widely used metrics, including recall (rec), precision (pre), accuracy (ACC), gain, and Matthews correlation coefficient (MCC). Compared to MSIsensor, scMSI can effectively reduce the false negative and false positive errors in detection and shows strong robustness when the sequencing depth and the number of clones change.

Further, scMSI is applied to the real dataset, a cohort of 16 endometrial cancer patients with clinical MSI status reported by immunohistochemistry but with negative MSI status from classical sequencing-based approaches. scMSI reported MSI on sub-clones successfully, and the findings matched the conclusions on immunohistochemistry. We also compare scMSI with MSIsnesor, MSIsensor-pro, mSINGS and MANTIS on the real dataset. Results demonstrated the effectiveness and superiority of scMSI in detecting MSI on sub-clones over existing approaches.

### 3.1. Study on simulation datasets

To evaluate the performance of scMSI in detecting the microsatellite status of each subclone, we conducted a series of simulation experiments with varying configurations, including different clone proportions, clone numbers, sequencing depths, and the distribution density of microsatellite lengths among clones. The number of microsatellites was set to be 60, and the read-length was set to be 200bps. The distribution parameters of microsatellite lengths in each clone were randomly chosen. The simulated data were also designed to include fully MSS samples for the evaluation of the false-positive errors.

a) Proportion of the primary clone

The proportion of primary clone in tumor sample was set to be 0.9, 0.7, 0.5, respectively. The coverage was set to be 200x. Comparing the performance of scMSI with MSIsensor, results summarized in Table 1 showed that as the proportion of the primary clone decreased, scMSI exhibited a slight increase in false positives but overall performed better than MSIsensor.

b) Sequencing depth

To explore the impact of different sequencing depths on the performance of scMSI, we set the number of microsatellites in each group to 60, the number of clones to 3. Sequencing depths ranged from 100x to 500x. Results from Table 2 indicated that the performance of scMSI improved with increasing sequencing depth. scMSI outperforms MSIsensor in terms of precision and recall rate, indicating that the false positives and false negatives are lower.

c) Number of clones

The number of clones was gradually increased from 3 to 5 with the sequencing depth set to be 800x. The experimental results are summarized in Table 3. The performance of scMSI decreased with a higher number of clones, as the detection difficulty increased when more clones were present.

d) Overlap in subclone length distributions

We set the sequencing depth to be 500x, 600x, 800x, and the number of clones to be 2, 3, and 4, correspondingly. When the overlapping degree of each component gradually increases, its influence on the detection performance is observed. These experimental results are presented in **Appendix B in S1 Text.** Increased overlap in microsatellite length distributions among subclones resulted in a performance decline, particularly when the overlap was significant, making the detection of microsatellite status more challenging for scMSI.

**Table 1:** Comparison results of scMSI and MSIsensor for different proportions of primary clones.

PC	scMSI					MSIsensor				
	Acc	Pre	Rec	MCC	Gain	Acc	Pre	Rec	MCC	Gain
0.9	0.950	0.909	1	0.904	0.900	0.633	0.580	0.967	0.358	0.267
0.7	0.933	0.882	1	0.874	0.867	0.620	0.570	0.967	0.330	0.233
0.5	0.900	0.833	1	0.816	0.800	0.517	0.509	0.933	0.060	0.033

**Table 2:** Performance comparison of scMSI and MSIsensor in different sequencing depths.

Depth	scMSI					MSIsensor				
	Acc	Pre	Rec	MCC	Gain	Acc	Pre	Rec	MCC	Gain
100X	0.817	0.744	0.967	0.675	0.633	0.433	0.463	0.833	-0.222	-0.133
300X	0.900	0.853	0.967	0.807	0.800	0.367	0.420	0.700	-0.358	-0.267
500X	0.933	0.882	1	0.874	0.867	0.467	0.481	0.867	-0.111	-0.067

**Table 3:** Comparison results of scMSI and MSIsensor for different clone numbers.

Number of clones	scMSI					MSIsensor				
	Acc	Pre	Rec	MCC	Gain	Acc	Pre	Rec	MCC	Gain
3	0.967	0.938	1	0.935	0.933	0.483	0.491	0.933	-0.094	-0.033
4	0.950	0.909	1	0.905	0.900	0.433	0.463	0.833	-0.243	-0.133
5	0.917	0.857	1	0.845	0.833	0.450	0.472	0.833	-0.156	-0.100

From the experimental results, compared with MSIsensor, scMSI can effectively reduce the false negative and false positive errors in microsatellite status detection. and the overall performance of scMSI is better than MSIsensor. Furthermore, our method also works well to obtain the true distribution of clonal microsatellite lengths. In the case of a large number of clones, our model can also well judge the status of microsatellites in each clone, while existing MSI detection algorithms cannot do this. Through multiple sets of simulation experiments, it can be found that the increasing number of clones will increase the detection difficulty of this method. As the number of clusters to which the microsatellite length distribution in clones belongs increases, the detection effect of this method is more dependent on the larger sequencing depth. For the distribution of microsatellite lengths in clones with a large degree of overlap, this method is more difficult to detect. With the increase in the density of microsatellite length distribution in each clone, the detection effect of the algorithm will decrease. And these factors that have an impact on our algorithm are also aspects that we would like to further improve in future research. Taken together, scMSI is superior to MSIsensor in all evaluation indicators for the clonal microsatellites status detection. The details of experiments design and results are presented in **Appendix B in S1 Text**.

### 3.2. Performance on the patient cohort dataset

To quantitatively describe the status of clonal microsatellites in each sample and evaluate the performance of the scMSI model, we first obtained microsatellite length distribution information and clonal structure information from 16 endometrial cancer patients. We then applied the scMSI model to the detection of sample clonal microsatellite states based on clonal structural information. As previously described, the immunohistochemical results of these 16 endometrial cancer samples showed loss of expression of at least one MMR protein, and the microsatellites in these samples were all MSI status. Compared to the IHC results of the corresponding samples, the scMSI results are completely consistent with the IHC results. To further illustrate the performance of the scMSI model, we also performed experiments on a patient cohort using MSIsnesor, MSIsensor-pro, mSINGS and MANTIS. The relevant test results are listed in Table 4.

**Table 4:** Comparison of test results among scMSI, MSIsensor, MSIsensor-pro, mSINGS, MANTIS and IHC for the patient cohort.

		MMR-IHC (Loss of Expression of.≥1 MMR Protein)
	
**scMSI**	MSI	16
	MSS	0
**MSIsensor**	MSI	7
	MSS	9
**MSIsensor-pro**	MSI	6
	MSS	10
**MSINGS**	MSI	2
	MSS	14
**MANTIS**	MSI	1
	MSS	14
	*No report	1

**Fig 4:**
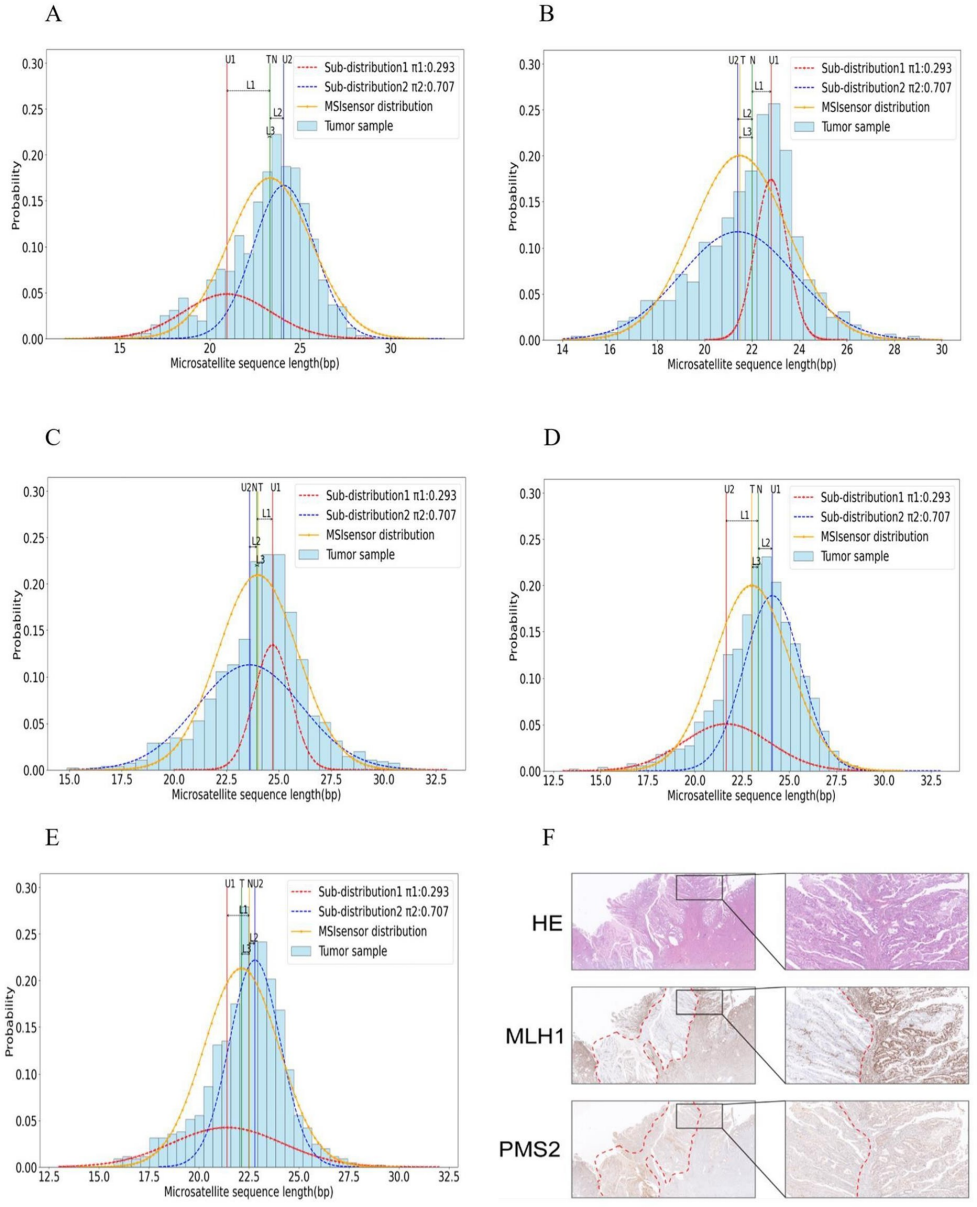
IHC detection map and PCR microsatellite detection site length distribution map of case 1. (A) BAT26 microsatellite length distribution map (B) NR24 microsatellite length distribution map (C) BAT25 microsatellite length distribution map (D) NR27 microsatellite length distribution map (E) NR21 microsatellite length distribution map (F) The figures of clonal immunohistochemical loss of MMR expression in tumor cells: clonal loss of MLH1 and PMS2 expression in the majority of tumor cells.

**Fig 5:**
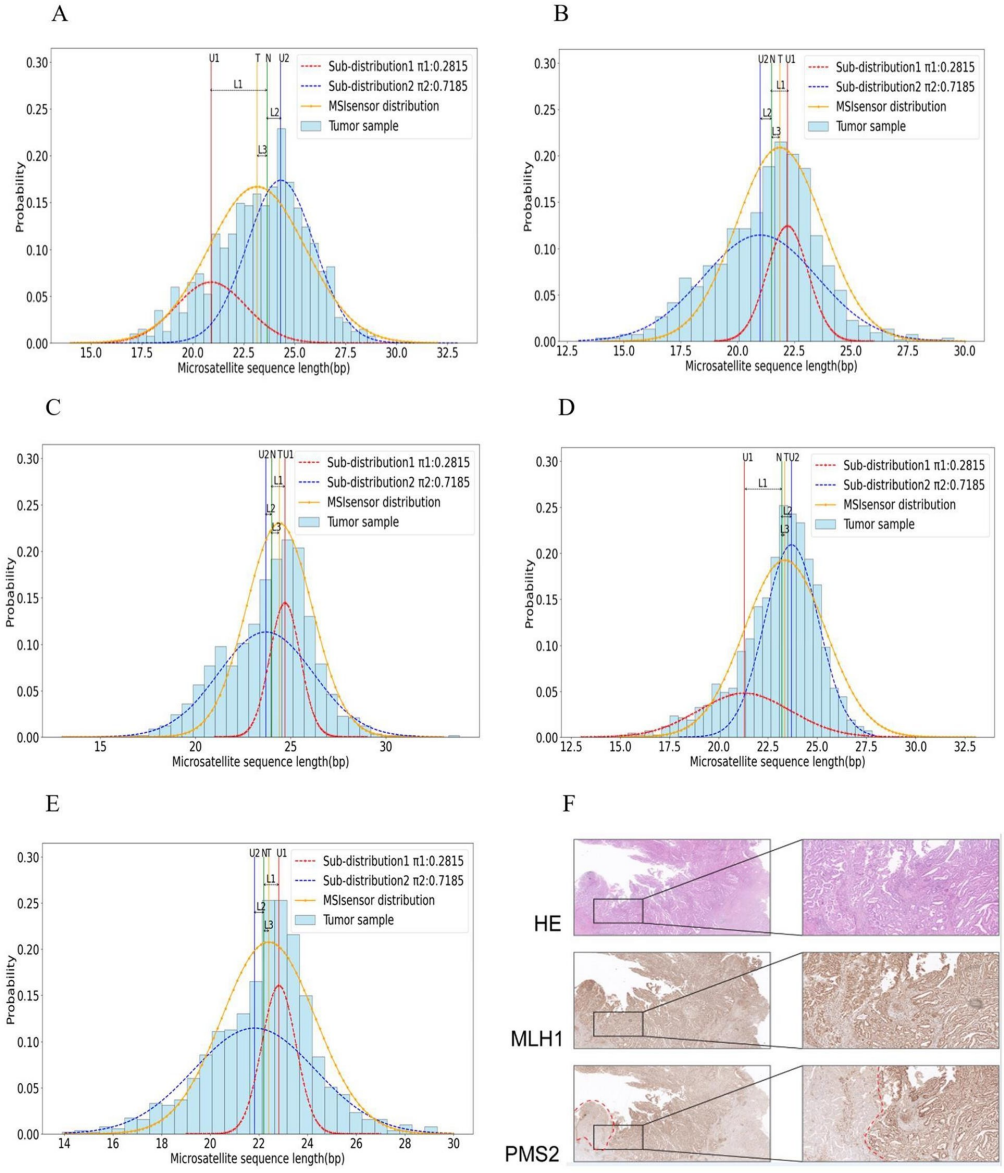
IHC detection map and PCR microsatellite detection site length distribution map of case 2. (A) BAT26 microsatellite length distribution map (B) NR24 microsatellite length distribution map (C) BAT25 microsatellite length distribution map (D) NR27 microsatellite length distribution map (E) NR21 microsatellite length distribution map (F) The figures of clonal immunohistochemical loss of MMR expression in tumor cells: clonal loss of PMS2 protein alone in focal tumor cells.

We selected 2 representative cases from 16 samples to describe scMSI performance in detail. MLH1 and PMS2 protein expression were absent in most tumor cells of case 1, and PMS2 protein expression was absent in tumor cells of case 2. The immunohistochemical results of each case are shown in Table 5. For these three samples, we summarized the scMSI clonal microsatellite status detection results and the MSIsensor, MSIsensor-pro, mSINGS and MANTIS microsatellite detection results in Tables 6 and 7. In order to better illustrate the clonal microsatellite status of each sample, we plotted the length distribution of clonal microsatellites for the PCR detection sites of each sample. And we attached the IHC detection result map of the corresponding sample to further illustrate the lack of cloned MMR expression in the tumor cells of the sample. The corresponding IHC detection result of samples and the length distribution of cloned microsatellites at PCR detection sites are shown in **Figs 4** and **5**. For the figures of clonal immunohistochemical loss of MMR expression in a variable proportion of tumor cells, Hematoxylin and eosin (HE) staining (top panel), MLH1 immunostaining (middle panel), and PMS2 immunostaining (low panel) figures in both low magnification (left) and intermediate magnification (right) are shown. And the red dotted line marked area is indicated as the loss of this expression region. The experimental results for the remaining samples are presented in **Appendix C in S1 Text**.

**Table 5:** Cases with clonal MMR deficiencies.

	MMR IHC Abnormality			
Case	MLH1	PMS2	MSH2	MSH6
1	−	−	+	+
2	+	−	+	+

**Table 6:** Detection of clonal microsatellite status for case 1.

Clone proportion	Site	μ	σ	π	Normal	MS	MSIsensor	MSIsensor-pro	MSINGS
[0.707, 0.293]	BAT26	20.95	2.38	0.293	23.33	MSI	MSS	MSS	MSS
		24.09	1.69	0.707	2.52	MSI			
	NR24	21.43	2.13	0.707	22.02	MSI	MSS	MSS	MSS
		22.80	0.67	0.293	1.85	MSI			
	BAT25	23.69	2.16	0.707	23.96	MSS	MSS	MSS	MSS
		24.65	0.87	0.293	2.34	MSI			
	NR27	24.07	1.49	0.707	23.36	MSI	MSS	MSS	MSS
		21.65	2.26	0.293	2.07	MSI			
	NR21	22.77	1.27	0.707	22.51	MSS	MSS	MSS	MSS
		21.49	2.76	0.293	1.75	MSI			
	MS1	12.62	1.33	0.293	13.49	MSI	MSS	MSS	MSS
		13.89	0.57	0.707	1.06	MSI			
	MS4				12.91		MSS	MSS	MSS
		12.89	0.81	1	0.74	MSS			
	MS5	16.7	1.6	0.293	17.69	MSI	MSS	MSS	MSS
		17.68	0.78	0.707	1.14	MSS			
	MS8				15.79		MSS	MSS	MSS
		15.77	1.04	1	1.01	MSS			
	MS9	15.92	0.69	0.707	15.68	MSS	MSS	MSS	MSS
		15.2	1.62	0.293	1.22	MSI			
	MS10				13.75		MSS	MSS	MSS
		13.74	0.85	1	0.85	MSS			
	MS12	13.77	1.32	0.293	15.39	MSI	MSS	MSS	MSS
		15.75	0.86	0.707	1.28	MSI			
	MS15				18.04		MSS	MSS	MSS
		18.13	1.24	1	1.22	MSS			
	MS22	16.33	1.42	0.707	16.57	MSS	MSS	MSS	MSS
		17.15	1.41	0.293	1.46	MSI			
	MS23	17.35	0.95	0.707	17.20	MSS	MSS	MSS	MSS
		16.83	2.2	0.293	1.46	MSI			
Sample							MSS	MSS	MSS
MANTIS	MSS								
IHC	partial MSI								

**Table 7:** Detection of clonal microsatellite status for case 2.

Clone proportion	Site	μ	σ	π	Normal	MS	MSIsensor	MSIsensor-pro	MSINGS
[0.7185, 0.2815]	BAT26	24.32	1.65	0.7185	23.66	MSI	MSS	MSS	MSS
		20.9	1.72	0.2815	2.54	MSI			
	NR24	21.17	2.23	0.7185	21.50	MSI	MSS	MSS	MSS
		22.2	0.9	0.2815	1.88	MSI			
	BAT25	24.65	0.86	0.2815	24.06	MSI	MSS	MSS	MSS
		23.71	2.15	0.7185	1.88	MSI			
	NR27	23.65	1.37	0.7185	23.22	MSI	MSS	MSS	MSS
		21.37	2.34	0.2815	1.88	MSI			
	NR21	21.82	2.12	0.7185	22.25	MSI	MSS	MSS	MSS
		22.82	0.75	0.2815	1.62	MSI			
	MS1	13.96	0.54	0.7185	13.61	MSI	MSS	MSS	MSS
		12.89	1.40	0.2815	1.09	MSI			
	MS4	12.78	1.12	0.2815	12.93	MSI	MSS	MSS	MSS
		12.97	0.47	0.7185	0.72	MSS			
	MS5				17.65		MSS	MSS	MSS
		17.56	1.09	1	1.10	MSS			
	MS8	16.38	0.84	0.7185	16.24	MSI	MSS	MSS	MSS
		15.86	1.71	0.2815	1.17	MSI			
	MS9				15.77		MSS	MSS	MSS
		15.76	1.09	1	1.18	MSS			
	MS10	14.32	1.57	0.2815	14.78	MSI	MSS	MSS	MSS
		14.94	0.54	0.7185	0.99	MSI			
	MS12	15.63	0.88	0.7185	15.21	MSI	MSS	MSS	MSS
		14.08	1.72	0.2815	1.32	MSI			
	MS15				18.0		MSS	MSS	MSS
		17.89	1.17	1	1.21	MSS			
	MS22	16.75	1.29	0.7185	16.61	MSS	MSS	MSS	MSS
		15.98	1.7	0.2815	1.52	MSI			
	MS23	18.47	1.17	0.7185	18.18	MSI	MSS	MSS	MSS
		16.79	2.11	0.2815	1.41	MSI			
Sample							MSS	MSS	MSS
MANTIS	MSS								
IHC	partial MSI								

According to these results, scMSI well distinguishs the status of sub-clonal MSI-events, while the results of MSI status are consistent with IHC reports. MSIsensor, MSIsensor-pro, mSINGS and MANTIS misjudged the microsatellite status of these three samples as MSS. Because MSIsensor and MSIsensor-pro algorithms are mainly divided into two steps, first, they determine the length distribution of microsatellite sequences, then they determine the status of microsatellites through statistical testing. In the first step of such methods, the microsatellite length distribution has been regarded as a unimodal distribution, and all clonal microsatellite states have been classified as a unique state. However, the length distribution of microsatellites in actual tumors is convoluted by the length distribution of multiple cloned microsatellites. These cloned microsatellites may have polymorphism, and the signals of these cloned microsatellites interfere with each other. When the length distribution of convolution is very similar to the reference length distribution, this will cause the existing algorithms to treat the microsatellite length distribution of the two samples as a consistent state in the detection process, thus introducing a false negative error. scMSI can avoid this problem and distinguish the state of each clone microsatellite well. For the mutation burden methods, purity, variant allelic frequency or sub-clonal proportions is not incorporated into the model via either the input, the structure or components of the pre-trained models. Compared with MSIsensor, MSIsensor-pro, mSINGS and MANTIS, scMSI shows higher accuracy and robustness on real datasets.

## 4. Conclusion

In this paper, we focus on the detection of sub-clonal microsatellite status on tumor-normal paired sequencing data. It is with important clinical implication to analysis the clonality of MSI events, but the existing approaches ignore the heterogeneity of cancer. We pointed out the computational challenges raised by considering multiple sub-clones, and developed an alternate iterative Bayesian model, namded scMSI, to overcome the difficulties. With synergistic integration of statistical learning and mathematical optimization, scMSI infers the allele distribution of microsatellites in clones so as to obtain the length distribution and status of microsatellites in each clone. A series of experiments on both real cohort and simulation datasets demonstrated the advantages of scMSI comparing to MSIsensor, MSIsensor-Pro, mSINGS and MANTIS on a wide range of settings. Specifically, scMSI effectively reduces the false positive and false negative errors in partial MSI-positive samples. It provides a new way of detecting MSI for cancers with a high degree of heterogeneity.

**Key points**

- scMSI is among the first computational approach to identify sub-clonal MSI events and to clearly report partial MSI-positive status.
- We released the following data: sequencing data of 16 endometrial cancer samples and the corresponding pathological images of immunohistochemically stained for mismatch repair (MMR) proteins. This is among the first dataset with paired sequencing data and pathological images on sub-clonal MSI-positive samples.
- scMSI is released and friendly to users with medical background.

## Supporting information

S1 Text Table A. Comparison results of scMSI and MSIsensor for different proportions of primary clones. Table B. Performance comparison of scMSI and MSIsensor in different sequencing depths. Table C. Comparison results of scMSI and MSIsensor for different clone numbers. Table D. When the number of subclones is 2 the detection results under different density distributions of microsatellite lengths in subclones at all levels. Table E. When the number of subclones is 3, the detection results under different density distributions of microsatellite lengths in subclones at all levels. Table F. When the number of subclones is 4, the detection results under different density distributions of microsatellite lengths in subclones at all levels. Table G. Cases with clonal MMR deficiencies. Table H. 15 microsatellite sites for detection. Table I. Classification of clonal microsatellite status for case 3. Table G. Classification of clonal microsatellite status for case 4. Table K. Classification of clonal microsatellite status for case 5. Table L. Classification of clonal microsatellite status for case 6. Table M. Classification of clonal microsatellite status for case 7. Table N. Classification of clonal microsatellite status for case 8. Table O. Classification of clonal microsatellite status for case 9. Table P. Classification of clonal microsatellite status for case 10. Table Q. Classification of clonal microsatellite status for case 11. Table R. Classification of clonal microsatellite status for case 12. Table S. Classification of clonal microsatellite status for case 13. Table T. Classification of clonal microsatellite status for case 14. Table U. Classification of clonal microsatellite status for case 15. Table V. Classification of clonal microsatellite status for case 16. **Fig A.** IHC detection map and PCR microsatellite detection site length distribution map of case 3. (A) BAT26 microsatellite length distribution map (B) NR24 microsatellite length distribution map (C) BAT25 microsatellite length distribution map (D) NR27 microsatellite length distribution map (E) NR21 microsatellite length distribution map. (F) The figures of clonal immunohistochemical loss of MMR expression in tumor cells: clonal loss of MLH1 and PMS2 expression in approximately half of the tumor cells. **Fig B.** IHC detection map and PCR microsatellite detection site length distribution map of case 4. (A) BAT26 microsatellite length distribution map (B) NR24 microsatellite length distribution map (C) BAT25 microsatellite length distribution map (D) NR27 microsatellite length distribution map (E) NR21 microsatellite length distribution map. (F) The figures of clonal immunohistochemical loss of MMR expression in tumor cells: clonal loss of MLH1 and PMS2 expression in approximately half of the tumor cells. **Fig C.** IHC detection map and PCR microsatellite detection site length distribution map of case 5. (A) BAT26 microsatellite length distribution map (B) NR24 microsatellite length distribution map (C) BAT25 microsatellite length distribution map (D) NR27 microsatellite length distribution map (E) NR21 microsatellite length distribution map. (F) The figures of clonal immunohistochemical loss of MMR expression in tumor cells: clonal loss of MLH1 and PMS2 protein in focal tumor cells. **Fig D.** IHC detection map and PCR microsatellite detection site length distribution map of case 6. (A) BAT26 microsatellite length distribution map (B) NR24 microsatellite length distribution map (C) BAT25 microsatellite length distribution map (D) NR27 microsatellite length distribution map (E) NR21 microsatellite length distribution map. (F) The figures of clonal immunohistochemical loss of MMR expression in tumor cells: clonal loss of MLH1 and PMS2 protein in the majority of tumor cells. **Fig E.** IHC detection map and PCR microsatellite detection site length distribution map of case 7. (A) BAT26 microsatellite length distribution map (B) NR24 microsatellite length distribution map (C) BAT25 microsatellite length distribution map (D) NR27 microsatellite length distribution map (E) NR21 microsatellite length distribution map. (F) The figures of clonal immunohistochemical loss of MMR expression in tumor cells: clonal loss of MLH1 and PMS2 protein in approximately half of the tumor cells. **Fig F.** IHC detection map and PCR microsatellite detection site length distribution map of case 8. (A) BAT26 microsatellite length distribution map (B) NR24 microsatellite length distribution map (C) BAT25 microsatellite length distribution map (D) NR27 microsatellite length distribution map (E) NR21 microsatellite length distribution map. (F) The figures of clonal immunohistochemical loss of MMR expression in tumor cells: clonal loss of MLH1 and PMS2 protein in approximately half of the tumor cells. **Fig G.** IHC detection map and PCR microsatellite detection site length distribution map of case 9. (A) BAT26 microsatellite length distribution map (B) NR24 microsatellite length distribution map (C) BAT25 microsatellite length distribution map (D) NR27 microsatellite length distribution map (E) NR21 microsatellite length distribution map. (F) The figures of clonal immunohistochemical loss of MMR expression in tumor cells: clonal loss of MSH2 and MSH6 protein in the majority of tumor cells. **Fig H.** IHC detection map and PCR microsatellite detection site length distribution map of case 10. (A) BAT26 microsatellite length distribution map (B) NR24 microsatellite length distribution map (C) BAT25 microsatellite length distribution map (D) NR27 microsatellite length distribution map (E) NR21 microsatellite length distribution map. (F) The figures of clonal immunohistochemical loss of MMR expression in tumor cells: clonal loss of MLH1 and PMS2 protein in the majority of tumor cells. **Fig I.** IHC detection map and PCR microsatellite detection site length distribution map of case 11. (A) BAT26 microsatellite length distribution map (B) NR24 microsatellite length distribution map (C) BAT25 microsatellite length distribution map (D) NR27 microsatellite length distribution map (E) NR21 microsatellite length distribution map. (F) The figures of clonal immunohistochemical loss of MMR expression in tumor cells: clonal loss of MLH1 and PMS2 protein in the majority of tumor cells. **Fig G.** IHC detection map and PCR microsatellite detection site length distribution map of case 12. (A) BAT26 microsatellite length distribution map (B) NR24 microsatellite length distribution map (C) BAT25 microsatellite length distribution map (D) NR27 microsatellite length distribution map (E) NR21 microsatellite length distribution map. (F) The figures of clonal immunohistochemical loss of MMR expression in tumor cells: clonal loss of MLH1 and PMS2 protein in focal tumor cells. **Fig K.** IHC detection map and PCR microsatellite detection site length distribution map of case 13. (A) BAT26 microsatellite length distribution map (B) NR24 microsatellite length distribution map (C) BAT25 microsatellite length distribution map (D) NR27 microsatellite length distribution map (E) NR21 microsatellite length distribution map. (F) The figures of clonal immunohistochemical loss of MMR expression in tumor cells: clonal loss of MLH1 and PMS2 protein in the majority of tumor cells. **Fig L.** IHC detection map and PCR microsatellite detection site length distribution map of case 14. (A) BAT26 microsatellite length distribution map (B) NR24 microsatellite length distribution map (C) BAT25 microsatellite length distribution map (D) NR27 microsatellite length distribution map (E) NR21 microsatellite length distribution map. (F) The figures of clonal immunohistochemical loss of MMR expression in tumor cells: clonal loss of MSH2 and MSH6 protein in the majority of tumor cells. **Fig M.** IHC detection map and PCR microsatellite detection site length distribution map of case 15. (A) BAT26 microsatellite length distribution map (B) NR24 microsatellite length distribution map (C) BAT25 microsatellite length distribution map (D) NR27 microsatellite length distribution map (E) NR21 microsatellite length distribution map. (F) The figures of clonal immunohistochemical loss of MMR expression in tumor cells: clonal loss of MSH2 and MSH6 protein in focal tumor cells. **Fig N.** IHC detection map and PCR microsatellite detection site length distribution map of case 16. (A) BAT26 microsatellite length distribution map (B) NR24 microsatellite length distribution map (C) BAT25 microsatellite length distribution map (D) NR27 microsatellite length distribution map (E) NR21 microsatellite length distribution map. (F) The figures of clonal immunohistochemical loss of MMR expression in tumor cells: clonal loss of MSH2 and MSH6 protein in focal tumor cells.
(PDF)
