# Supplementary material for: scMSI: Accurately inferring the sub-clonal Micro-Satellite status by an integrated deconvolution model on length spectrum
Source: PLoS Comput Biol. 2024 Dec 2;20(12):e1012608. doi: 10.1371/journal.pcbi.1012608 (PMC11637434; doi:10.1371/journal.pcbi.1012608)
Supplement: S1 Text — Table A. Comparison results of scMSI and MSIsensor for different proportions of primary clones. Table B. Performance comparison of scMSI and MSIsensor in different sequencing depths. Table C. Comparison results of scMSI and MSIsensor for different clone numbers. Table D. When the number of subclones is 2 the detection results under different density distributions of microsatellite lengths in subclones at all levels. Table E. When the number of subclones is 3, the detection results under different density distributions of microsatellite lengths in subclones at all levels. Table F. When the number of subclones is 4, the detection results under different density distributions of microsatellite lengths in subclones at all levels. Table G. Cases with clonal MMR deficiencies. Table H. 15 microsatellite sites for detection. Table I. Classification of clonal microsatellite status for case 3. Table G. Classification of clonal microsatellite status for case 4. Table K. Classification of clonal microsatellite status for case 5. Table L. Classification of clonal microsatellite status for case 6. Table M. Classification of clonal microsatellite status for case 7. Table N. Classification of clonal microsatellite status for case 8. Table O. Classification of clonal microsatellite status for case 9. Table P. Classification of clonal microsatellite status for case 10. Table Q. Classification of clonal microsatellite status for case 11. Table R. Classification of clonal microsatellite status for case 12. Table S. Classification of clonal microsatellite status for case 13. Table T. Classification of clonal microsatellite status for case 14. Table U. Classification of clonal microsatellite status for case 15. Table V. Classification of clonal microsatellite status for case 16. Fig A. IHC detection map and PCR microsatellite detection site length distribution map of case 3. (A) BAT26 microsatellite length distribution map (B) NR24 microsatellite length distribution map (C) BAT25 microsa [file pcbi.1012608.s001.pdf]

Supporting information for

scMSI: accurately inferring the sub-clonal Micro-Satellite Status by an integrated deconvolution model on length spectrum

Yuqian Liu[1,#], Yan Chen[1,#], Huanwen Wu[2,#], Xuanping Zhang[1], Yuqi Wang[1,3], Xin Yi[3], Zhiyong Liang[2,*] and Jiayin Wang[1,*]

[1]School of Computer Science and Technology, Xi'an Jiaotong University, Xi'an, China
[2]Department of Pathology, State Key Laboratory of Complex Severe and Rare Disease, Molecular Pathology Research Center, Peking Union Medical College Hospital, Chinese Academy of Medical Sciences and Peking Union Medical College, Beijing, China.
[3]Geneplus Beijing Institute, Beijing, China.
[#]These authors have contributed equally to this work and share first authorship

* Correspondence:

wangjiayin@mail.xjtu.edu.cn;

liangzy@pumch.cn

Appendix A. Clonal microsatellite state detection algorithm

We employ an alternate iterative strategy to obtain clonal microsatellite states. In the process of updating the approximate distribution of potential variable Z, the logarithm form of the approximate posterior distribution of the latent variable Z can be obtained as

$$\mathrm{In}q(z) = E_{\pi,\mu,\Lambda}[\mathrm{In}P(\mathbf{L}, Z, \pi, \mu, \Lambda)]$$

$$= \sum_{n=1}^{N}\sum_{k=1}^{K} z_{nk}[E_{\pi_k}[\mathrm{In}\pi_k] + \frac{1}{2}E_{\Lambda_k}[\mathrm{In}|\Lambda_k|]$$

$$-\frac{D}{2}\mathrm{In}(2\pi)$$

$$-\frac{1}{2}E_{\mu_k\Lambda_k,}[(\mathbf{L}_n - \mu_k)^T \Lambda_k (\mathbf{L}_n - \mu_k)] \quad (1)$$

We can get the following optimal solution:

$$q(z) = \prod_{n=1}^{N}\prod_{k=1}^{K} r_{nk}^{z_{nk}} \tag{2}$$

and

$$r_{nk} = \frac{\rho_{nk}}{\sum_{i=1}^{K}\rho_{ni}} \tag{3}$$

$$\rho_{nk} = \exp\left\{\frac{1}{2}\sum_{i=1}^{D}\Psi\left(\frac{v_k + 1 - i}{2}\right) + \frac{D}{2}\mathrm{In}2 + \frac{1}{2}\mathrm{In}|w_k| - \frac{v_k}{2}(L_n - m_k)^T w_k(\mathbf{L}_n - m_k) + \Psi(Y_k) - \frac{D}{2}\mathrm{In}(2\pi)\right.$$

$$\left. - \frac{D}{2}\beta_k^{-1} - \Psi\left(\sum_{k=1}^{K} Y_k\right)\right\} \tag{4}$$

Meanwhile, the probability distribution of variable $\mathbf{L}_n$ to cluster k can be provided through the posterior probability $p(z_{nk} = 1|\mathbf{L}_n) = r_{nk}$. If visualization is required, these probability assignments can also be converted into hard assignments, and $\mathbf{L}_n$ is assigned to one and only one cluster.

$$k = \mathrm{argmax}_{k'}\, p(z_{nk'} = 1|\mathbf{L}_n) \tag{5}$$

In the process of updating model parameters, the introduced equation (9) is an integer programming problem, which is an NP hard problem. When solving integer programming, the first thing that comes to mind is to enumerate the integer combinations of all feasible solutions of variables. For small-scale problems, the enumeration method is feasible, but for large-scale problems, the number of feasible integer combinations is quite large, which may lead to the combinatorial explosion problem. Obviously, the enumeration method is not desirable to solve such problems. The branch and bound method can reduce the calculation of the problem while ensuring the accuracy. It improves the efficiency of branch decision-making by eliminating a large number of nodes that have no hope of exceeding the known optimal solution, and finally obtains the optimal solution of the problem. The branch and bound method has a good application to the solution of integer programming and has the advantages of fast average speed and optimal solution.

Thus, we mainly use the branch and bound method to solve the introduced objective function. First, we set the combined proportion F_k of each clone can be expressed as

$$F_k = \sum_{g=1}^{G} \mathbf{B}_g A_{gk} \tag{6}$$

Without considering the variable integer constraints, we solve the optimal solution of the original problem corresponding to the relaxed problem

$$\min \sum_{n=1}^{N} \left\{ \sum_{k=1}^{K} F_k \frac{1}{\sqrt{2\pi\sigma_k^2}} \exp\left\{ -\frac{1}{2\sigma_k^2} (\mathbf{L}_n - \mu_k)^2 \right\} - f(\mathbf{L}_n) \right\}^2$$

$$\text{s.t. } 0 \le A_{gk} \le 1$$

$$\sum_{k=1}^{K} A_{gk} = 1 \tag{7}$$

If the optimal solution satisfies all integer constraints, the optimal solution of the original problem is obtained. If it is not satisfied, you need to branch to any of the variables, and add the two branched constraints to the relaxation problem Q_0, divide the original relaxation problem into two sub-problems Q_1 and Q_2, and then solve the two sub-problems separately. According to needs, each sub-problem can generate its own sub-problems again, thus generating a search tree, and the search space is also divided into smaller spaces. The original question Q_0 is the root node of the tree, the sub-questions generated in the search process are the nodes of the tree, and the leaf nodes are all nodes that have not yet branched.

In the process of searching, if the optimal solution of a node in the search tree satisfies all integer restrictions, the node is marked as searched, making it a permanent leaf node, and no further branching will be made afterwards. Then compare the objective function value of the optimal solution of the node with the current optimal objective function value H. If it is better than H (or there is no optimal objective function value currently), then the objective function value of the node is set to a new limit. If the objective function value of the optimal solution of the node is greater than or equal to the upper limit H, then the node will be pruned and will not be considered afterwards. The search area is reduced primarily by truncating regions containing invalid solutions without having to enumerate them. Then continue the search until the difference between the minimum value of the optimal objective function value of all current leaf nodes and the current upper limit is zero, indicating that the current upper bound is optimal. During the search process, different nodes are processed in parallel, which can greatly reduce the search time.

After obtaining the optimal solution A that meets the requirements, it is substituted into the update of the entire model parameters. After derivation, the update formula of the model parameters can be obtained.

$$\mathrm{Y_k} = \mathbf{B}_\mathrm{g} \mathrm{A_{gk}} \sum_{k=1}^{K} \mathrm{Y_k} \tag{8}$$

$$\beta_\mathrm{k} = \mathrm{N_k} + \beta_0 \tag{9}$$

$$\mathrm{m_k} = \frac{1}{\beta_\mathrm{k}} (\beta_0 \mathrm{m_0} + \mathrm{N_k x_k}) \tag{10}$$

$$\mathrm{v_k} = \mathrm{N_k} + \mathrm{v_0} \tag{11}$$

$$\mathrm{w_k^{-1}} = \mathrm{w_0^{-1}} + \mathrm{N_k S_k} + \frac{\beta_0 \mathrm{N_k}}{\beta_\mathrm{k}} (\mathrm{x_k} - \mathrm{m_0})(\mathrm{x_k} - \mathrm{m_0})^\mathrm{T} \tag{12}$$

and

$$\mathrm{N_k} = \sum_{\mathrm{n}=1}^{\mathrm{N}} \mathrm{r_{nk}} \tag{13}$$

$$\mathrm{x_k} = \frac{1}{\mathrm{N_k}} \sum_{\mathrm{n}=1}^{\mathrm{N}} \mathrm{r_{nk}}\, \mathbf{L}_\mathrm{n} \tag{14}$$

$$\mathrm{S_k} = \frac{1}{\mathrm{N_k}} \sum_{\mathrm{n}=1}^{\mathrm{N}} \mathrm{r_{nk}}\, (\mathbf{L}_\mathrm{n} - \mathrm{x_k})(\mathrm{L_n} - \mathrm{x_k})^\mathrm{T} \tag{15}$$

The iteration terminates until the difference between the adjacent lower bounds of evidence reaches a certain threshold. The length distribution parameters of microsatellites in each clone are obtained, and finally the status of clonal microsatellites is determined by statistical test. Moreover, for multiple microsatellite loci, our model also designs parallel deconvolution, utilizing multiple microsatellite loci for parallelized microsatellite state estimation to reduce the running time.

Appendix B. Simulation studies

To explore the properties of scMSI and evaluate its ability of detecting the microsatellite status of each subclone, we conducted experiments on a series of simulated datasets with different configurations by varying the proportion of clones, the number of clones, sequencing depth, and the distribution density of microsatellite lengths among clones.

a. Proportion of the primary clone

The number of microsatellites was set to be 60, the coverage was set to be 200× and the read-length was set to be 200bps. The proportion of primary clones in tumor was set to be 0.9, 0.7, 0.5, respectively. The distribution parameters of microsatellite lengths in each clone were randomly chosen. The scMSI and MSIsenosr results are summarized in **Table A**, where pc represents the proportion of primary clones.

Table A. Comparison results of scMSI and MSIsensor for different proportions of primary clones.

	scMSI					MSIsensor				
PC	Acc	Pre	Rec	MCC	Gain	Acc	Pre	Rec	MCC	Gain
0.9	0.950	0.909	1	0.904	0.900	0.633	0.580	0.967	0.358	0.267
0.7	0.933	0.882	1	0.874	0.867	0.620	0.570	0.967	0.330	0.233
0.5	0.900	0.833	1	0.816	0.800	0.517	0.509	0.933	0.060	0.033

It can be seen from **Table A** that as the proportion of the main clone in the tumor continues to decrease, the false positive of scMSI will slightly increase, and the overall performance will decrease. The false positives and false negatives of MSIsensor will increase with the decrease of the proportion of primary clones in the tumor. The overall performance of scMSI is better than that of MSIsensor.

b. Sequencing depth

Sequencing depth affects somatic mutation calling, which in turn may affect tumor heterogeneity. Therefore, the impact of sequencing depth on the performance of this algorithm also needs to be considered. To explore the impact of different sequencing depths on the performance of this algorithm, we set the number of microsatellites in each group to 60, the number of clones to 3. The sequencing depths were varied from

100x, 300x, and 500x. It can be seen from **Table B** that the change of sequencing depth will obviously affect the change of each index of this algorithm.

Table B. Performance comparison of scMSI and MSIsensor in different sequencing depths.

	scMSI					MSIsensor				
Depth	Acc	Pre	Rec	MCC	Gain	Acc	Pre	Rec	MCC	Gain
100X	0.817	0.744	0.967	0.675	0.633	0.433	0.463	0.833	-0.222	-0.133
300X	0.900	0.853	0.967	0.807	0.800	0.367	0.420	0.700	-0.358	-0.267
500X	0.933	0.882	1	0.874	0.867	0.467	0.481	0.867	-0.111	-0.067

From **Table B**, it can be seen that the performance of this algorithm in identifying microsatellite states and length distribution parameters in each clone increases gradually with the increase of sequencing depth. Mainly when the number of real components of the mixed distribution of microsatellites in the tumor is large, a higher sequencing depth can ensure that each component has more easily distinguishable length distribution data. This algorithm is better than MSIsensor in terms of precision and recall rate, indicating that the false positives and false negatives of our algorithm are lower than those of MSIsensor.

c. Number of clones

We also consider that the variation in the number of clones in the tumor will also be a factor affecting the performance of this algorithm. We set the sequencing depth to 800x and the number of microsatellites in each group to 60. The number of clones was gradually increased from 3 to 5 to analyze the effect of different numbers of clones on the performance of the algorithm. The experimental results are summarized in **Table C**.

Table C. Comparison results of scMSI and MSIsensor for different clone numbers.

	scMSI					MSIsensor				
Number of clones	Acc	Pre	Rec	MCC	Gain	Acc	Pre	Rec	MCC	Gain
3	0.967	0.938	1	0.935	0.933	0.483	0.491	0.933	-0.094	-0.033
4	0.950	0.909	1	0.905	0.900	0.433	0.463	0.833	-0.243	-0.133
5	0.917	0.857	1	0.845	0.833	0.450	0.472	0.833	-0.156	-0.100

It can be seen from **Table C** that with the increased number of clones, the performance of our algorithm decreases significantly. It can be seen that when the number of clones is small, since the number of clones is closer to the true number of components, it is easier for the algorithm to accurately identify the state and length distribution parameters of microsatellites in each clone. As the number of clones increases, the detection difficulty of the algorithm increases.

d. The degree of overlap among length distributions of subclones

The degree of overlap between components in the mixed distribution of microsatellites in tumors is an indicator that affects the performance of the algorithm. Therefore, we designed the sequencing depth to be 500x, 600x, 800x, and the number of clones corresponded to 2, 3, and 4, respectively. When the overlapping degree of each component gradually increases, its influence on the performance of this algorithm is observed. The experimental results are summarized in **Table D** to **Table F**. From the experimental results, it can be seen that when the overlap of each component gradually increases, the performance of the algorithm gradually decreases. This is mainly because the components with a small degree of overlap are more conducive to the scMSI algorithm to distinguish the status of clonal microsatellites.

Table D. When the number of subclones is 2 the detection results under different density distributions of microsatellite lengths in subclones at all levels.

Simulation data					Test Results				scMSI	MSIsensor
Number of samples	Subclone structural information	π	μ	σ	π'	μ'	σ'	MS state	MS state	MS state
500	M=2 [0.75,0.25]	0.75	24	1	0.75	24.03	1.03	MSI		
		0.25	20	1	0.25	20.09	1.23	MSI	MSI	MSI
		0.75	23	1	0.75	23.00	1.08	MSS		
		0.25	20	1	0.25	20.07	1.19	MSI	MSS	MSI
		0.75	22	1	0.75	22.04	1.14	MSS		
		0.25	20	1	0.25	20.24	1.01	MSI	MSS	MSI
		0.75	21	1	0.75	21.13	0.98	MSI		
		0.25	20	1	0.25	19.56	0.83	MSI	MSI	MSS
		0.75	23	1	0.75	23.45	1.09	MSS		
		0.25	24	1.3	0.25	22.36	0.89	MSI	MSS	MSS

Table E. When the number of subclones is 3, the detection results under different density distributions of microsatellite lengths in subclones at all levels.

Simulation data					Test Results				scMSI	MSIsensor
Number of samples	Subclone structural information	π	μ	σ	π'	μ'	σ'	MS state	MS state	MS state
600	M=3 [0.906, 0.093, 0.001]	0.907	23.84	1.2	0.907	23.81	1.29	MSI	MSI	MSS
		0.093	20.34	1.4	0.093	20.46	1.52	MSI		
		0.907	23.74	1.2	0.907	23.73	1.29	MSI	MSI	MSS
		0.093	20.74	1.4	0.093	21.00	1.69	MSI		
		0.907	23.74	1.4	0.907	23.67	1.42	MSI	MSI	MSS
		0.093	21.24	1.4	0.093	21.41	1.68	MSI		
		0.907	23.45	1.4	0.906	23.58	1.39	MSI	MSI	MSI
		0.093	21.8	1.4	0.094	21.63	1.46	MSI		
		0.907	23.5	1.4	0.907	23.63	1.37	MSS	MSS	MSI
		0.093	22.5	1.4	0.093	21.71	1.26	MSI		

Table F. When the number of subclones is 4, the detection results under different density distributions of microsatellite lengths in subclones at all levels.

Simulation data					Test Results				scMSI	MSIsensor
Number of samples	Subclone structural information	π	μ	σ	π'	μ'	σ'	MS state	MS state	MS state
800	M=4 [0.69,0.16,0.1, 0.05]	0.69	9	1	0.69	8.98	1.06	MSS	MSS	MSI
		0.16	12	0.5	0.16	11.87	0.60	MSI		
		0.15	7	0.55	0.15	7.11	0.73	MSI		
		0.69	9	1	0.69	8.91	1.15	MSS	MSS	MSS
		0.16	11	0.5	0.16	11.00	0.69	MSI		
		0.15	7	0.55	0.15	7.31	0.85	MSI		
		0.69	9	1	0.69	9.04	1.02	MSS	MSS	MSS
		0.16	11	0.5	0.16	10.89	0.71	MSI		
		0.15	8	0.55	0.15	7.87	0.64	MSI		
		0.69	9	1	0.69	9.03	0.97	MSS		

		0.16	10	0.5	0.15	10.28	0.67	MSI	MSS	MSS
		0.15	7	0.55	0.16	6.99	0.60	MSI		
		0.16	10	0.5	0.15	9.92	0.67	MSI		
		0.69	9	1	0.69	8.90	1.05	MSS	MSS	MSS
		0.15	8	0.55	0.16	8.45	0.84	MSI		

From the experimental results of all simulation data, it can be seen that compared with MSIsensor, scMSI can effectively reduce the false negative and false positive errors in microsatellite status detection, and the overall performance of scMSI is better than MSIsensor. Furthermore, our method also works well to obtain the true distribution of clonal microsatellite lengths. In the case of a large number of clones, our model can also well judge the status of microsatellites in each clone, while existing MSI detection algorithms cannot do this. Through multiple sets of simulation experiments, it can be found that the increasing number of clones will increase the detection difficulty of this method. As the number of clusters to which the microsatellite length distribution in clones belongs increases, the detection effect of this method is more dependent on the larger sequencing depth. For the distribution of microsatellite lengths in clones with a large degree of overlap, this method is more difficult to detect. With the increase in the density of microsatellite length distribution in each clone, the detection effect of the algorithm will decrease. And these factors that have an impact on our algorithm are also aspects that we would like to further improve in future research. Taken together, our model has good state determination ability in clonal microsatellites.

Appendix C. Real data cohort experiment

We mainly performed experiments on a cohort of 16 patients to validate the performance of the scMSI model. The immunohistochemical results of the remaining 14 cases showed at least 2 MMR protein deletions, and the relevant immunohistochemical results are listed in the **Table G**. In addition, 15 microsatellite sites selected for status detection are listed in **Table H.**

Table G. Cases with clonal MMR deficiencies.

	MMR IHC Abnormality			
Case	MLH1	PMS2	MSH2	MSH6
3	−	−	+	+
4	−	−	+	+
5	−	−	+	+
6	−	−	+	+
7	−	−	+	+
8	−	−	+	+
9	+	+	−	−
10	−	−	+	+
11	−	−	+	+
12	−	−	+	+
13	−	−	+	+
14	+	+	−	−
15	+	+	−	−
16	+	+	−	−

Table H. 15 microsatellite sites for detection.

Locus identity	Chr	Position	Homopolymer	Left-mer	Right-mer
BAT26	2	47641559	27[A]	CAGGT	GGGTT
NR24	2	95849361	23[T]	TCCTA	GTGAG
BAT25	4	55598211	25[T]	TTTGA	GAGAA
NR27	11	102193508	26[A]	CTGGT	GCCAC

NR21	14	23652346	21[A]	TTGCT	GGCCA
MS1	1	161332091	14[T]	ATTCC	GCTTT
MS4	2	48032740	13[T]	TGTGA	AAGGT
MS5	2	48033890	18[T]	AAAC	AATTT
MS8	7	6037057	17[A]	AACTG	TTCAC
MS9	7	116381121	16[T]	TGGTG	GGTTT
MS10	7	116409675	15[T]	CAACC	CCTTT
MS12	11	108121410	15[T]	TATCC	AGGCT
MS15	11	108195976	19[T]	CATAG	CATTT
MS22	18	48584855	16[T]	GGCTA	GGTAG
MS23	2	39536689	27[T]	CAGGA	GAGGC

The homopolymer was described using the repeat length and the repeat unit. For example, MS15 is the locus in chromosome 11. The flanking sequences on the left and right side of the homopolymer are CATAG and CATTT, respectively.

Immunostaining results for MMR proteins in all the collected patient cohorts were analyzed and evaluated by two experienced pathologists (Wu H and Liang Z), and the remaining 14 cases were also MSI status. For these 14 cases, MSIsensor misjudged 7 of them as MSS status, while MSIsensor-Pro misjudged 8 of them as MSS status. Both MSINGS and MANTIS misjudged 12 cases as MSS status. The detection results of scMSI model are completely consistent with the results of IHC, and the state of each clone microsatellite can be well judged. The relevant experimental results of the remaining 14 cases are summarized as follows.

Table I. Classification of clonal microsatellite status for case 3.

Clone proportion	Site	μ	σ	π	Normal	MS	MSIsensor	MSIsensor-pro	MSINGS
[0.649, 0.351]	BAT26	24.13<br>21.65	1.79<br>3.21	0.649<br>0.351	23.25<br>2.71	MSI<br>MSI	MSS	MSS	MSS
	NR24	22.15<br>20.95	0.91<br>2.28	0.351<br>0.649	21.64<br>1.87	MSI<br>MSI	MSS	MSS	MSS
	BAT25	23.94<br>25.34	2.22<br>1.07	0.649<br>0.351	24.51<br>1.94	MSI<br>MSI	MSS	MSS	MSS
	NR27	24.59<br>21.74	1.27<br>2.06	0.649<br>0.351	23.67<br>2.10	MSI<br>MSI	MSS	MSS	MSS
	NR21	22.57<br>20.87	1.1<br>2.71	0.649<br>0.351	22.08<br>1.77	MSI<br>MSI	MSS	MSS	MSS
					13.59				

	MS1	13.61	1.01	1	1.04	MSS	MSS	MSS	MSS
	MS4	12.93	0.74	1	12.93<br>0.78	MSS	MSS	MSS	MSS
	MS5	17.02<br>17.9	1.34<br>0.73	0.351<br>0.649	17.62<br>1.19	MSI<br>MSI	MSS	MSS	MSS
	MS8	18.05<br>15.87	0.81<br>1.02	0.351<br>0.649	16.73<br>1.42	MSI<br>MSI	MSS	MSS	MSS
	MS9	15.72	1.33	1	15.83<br>1.14	MSS	MSS	MSS	MSS
	MS10	14.13<br>14.96	1.6<br>0.53	0.351<br>0.649	14.70<br>1.09	MSI<br>MSI	MSS	MSS	MSS
	MS12	14.58<br>15.42	1.54<br>0.7	0.649<br>0.351	14.91<br>1.33	MSI<br>MSI	MSS	MSS	MSS
	MS15	18.10	1.28	1	17.94<br>0.99	MSS	MSS	MSS	MSS
	MS22	15.82	1.23	1	15.73<br>1.05	MSS	MSS	MSS	MSS
	MS23	18.07	1.62	1	18.21<br>1.64	MSS	MSS	MSS	MSS
Sample							MSS	MSS	MSS
MANTIS								MSS	
IHC								partial MSI	

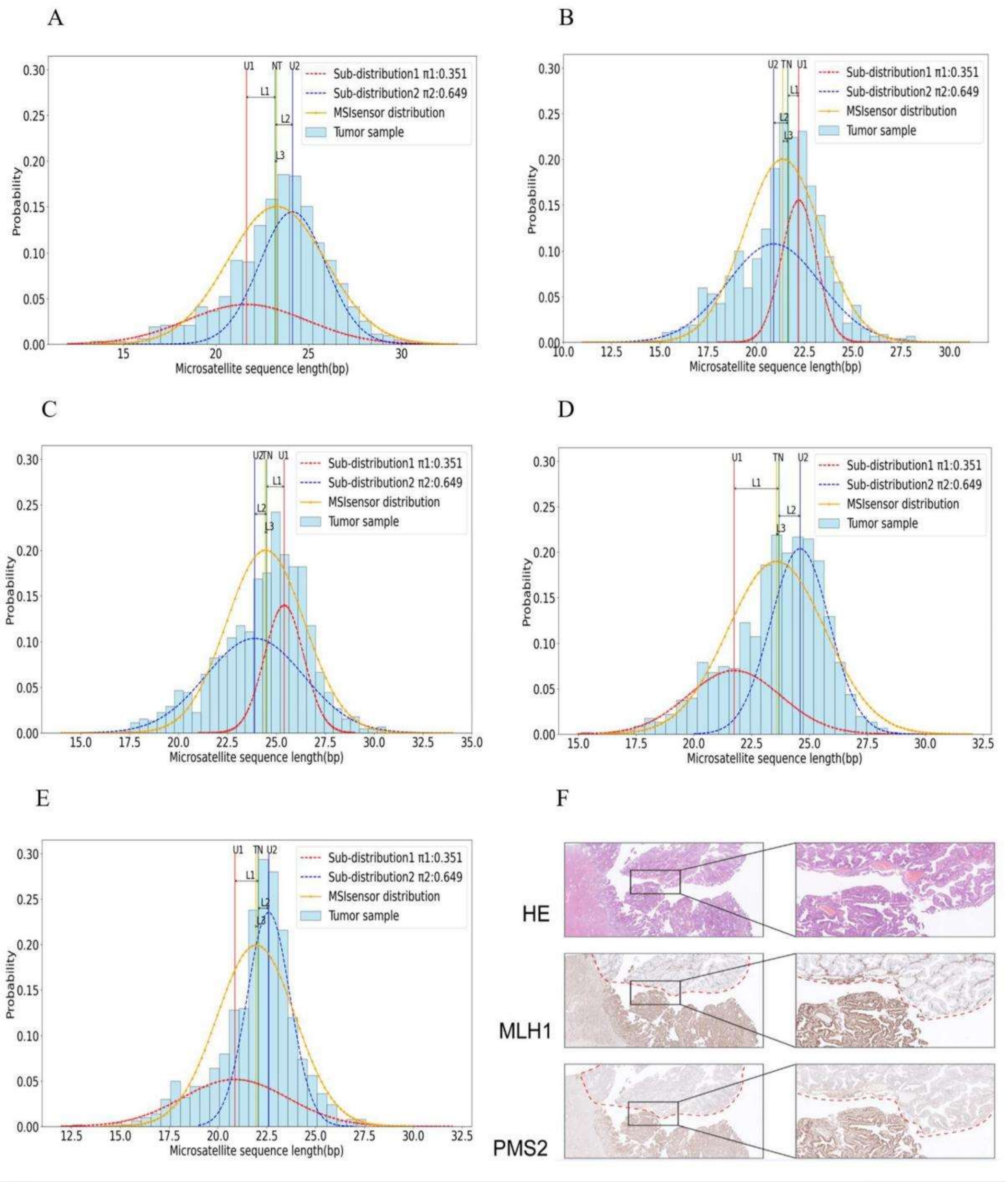

Figure A. IHC detection map and PCR microsatellite detection site length distribution map of case 3. (A) BAT26 microsatellite length distribution map (B) NR24 microsatellite length distribution map (C) BAT25 microsatellite length distribution map (D) NR27 microsatellite length distribution map (E) NR21 microsatellite length distribution map (F) The figures of clonal immunohistochemical loss of MMR expression in tumor cells: clonal loss of MLH1 and PMS2 expression in approximately half of the tumor cells.

Table J. Classification of clonal microsatellite status for case 4.

Clone proportion	Site	μ	σ	π	Normal	MS	MSIsensor	MSIsensor-pro	MSINGS
		21.70	2.85	0.326	23.31	MSI			
	BAT26	23.96	1.61	0.674	2.41	MSI	MSS	MSS	MSS
		22.47	1.14	0.674	22.11	MSI			

	NR24	21.12	2.67	0.326	1.92	MSI	MSS	MSS	MSS
[0.674,<br>0.326]	BAT25	24.17<br>22.04	1.04<br>2.38	0.674<br>0.326	23.68<br>2.09	MSI<br>MSI	MSS	MSS	MSS
	NR27	24.38<br>22.09	1.46<br>2.17	0.674<br>0.326	23.55<br>2.03	MSI<br>MSI	MSS	MSS	MSS
	NR21	21.38<br>22.96	2.3<br>1.26	0.326<br>0.674	22.65<br>1.87	MSI<br>MSI	MSS	MSS	MSS
	MS1	13.06<br>13.95	1.42<br>0.52	0.326<br>0.674	13.59<br>1.07	MSI<br>MSI	MSS	MSS	MSS
	MS4	12.97	0.73	1	12.92<br>0.75	MSS	MSS	MSS	MSS
	MS5	17.8<br>16.99	0.75<br>1.65	0.674<br>0.326	17.63<br>1.24	MSS<br>MSI	MSS	MSS	MSS
	MS8	16.05<br>18.09	1.13<br>0.81	0.674<br>0.326	16.66<br>1.49	MSI<br>MSI	MSS	MSS	MSS
	MS9	15.74	1.20	1	15.74<br>1.09	MSS	MSS	MSS	MSS
	MS10	14.78	0.99	1	14.67<br>1.02	MSI	MSS	MSS	MSS
	MS12	14.49<br>15.36	1.5<br>0.66	0.674<br>0.326	14.80<br>1.32	MSI<br>MSI	MSS	MSS	MSS
	MS15	17.99	1.22	1	18.00<br>1.10	MSS	MSS	MSS	MSS
	MS22	15.29<br>16.02	1.64<br>0.5	0.326<br>0.674	15.74<br>1.13	MSI<br>MSI	MSS	MSS	MSS
	MS23	17.45<br>17.91	1.94<br>1.15	0.674<br>0.326	17.59<br>1.70	MSS<br>MSI	MSS	MSS	MSS
Sample							MSS	MSS	MSS
MANTIS								MSS	
IHC								partial MSI	

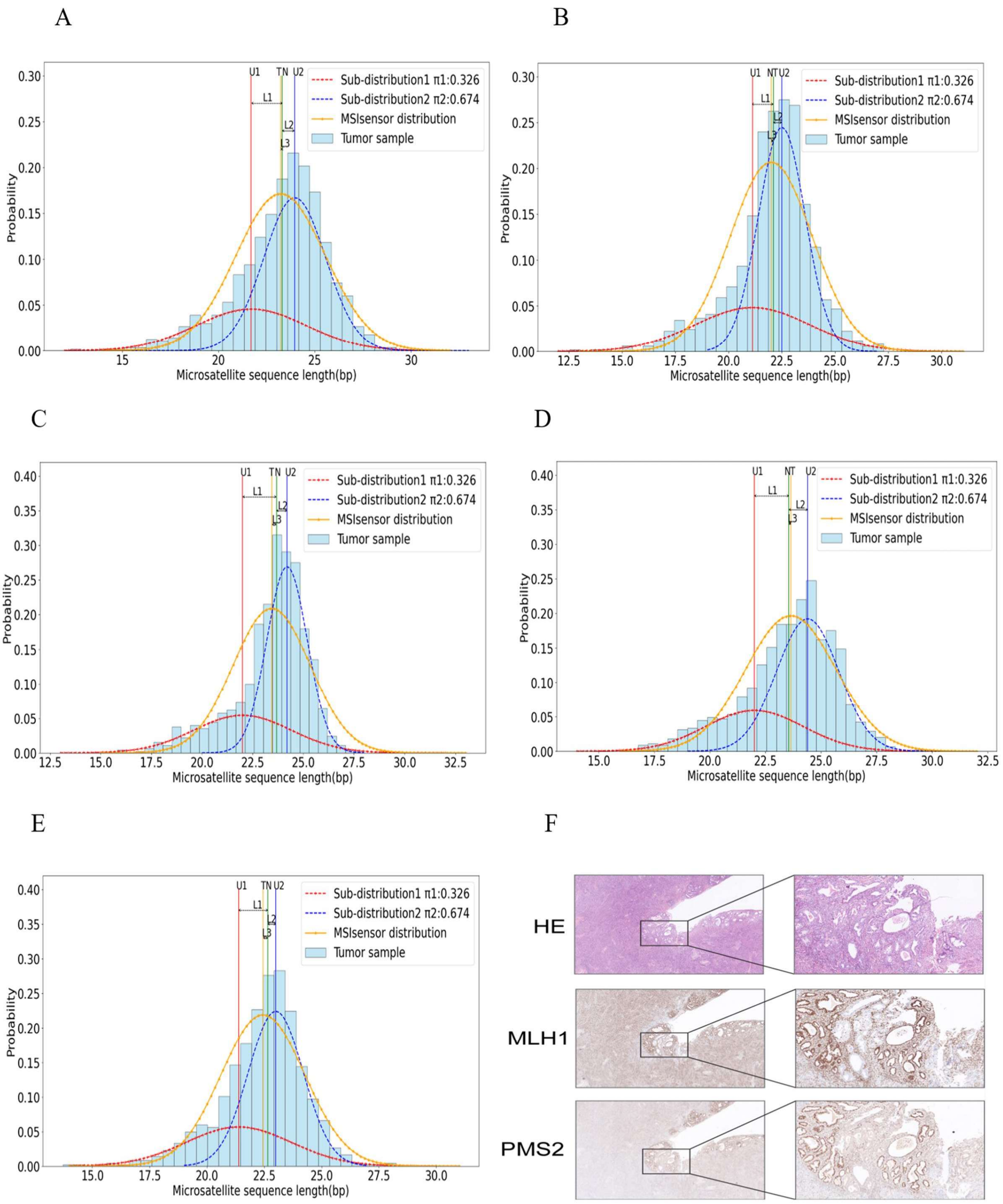

Figure B. IHC detection map and PCR microsatellite detection site length distribution map of case 4. **(A)** BAT26 microsatellite length distribution map **(B)** NR24 microsatellite length distribution map **(C)** BAT25 microsatellite length distribution map **(D)** NR27 microsatellite length distribution map **(E)** NR21 microsatellite length distribution map. **(F)** The figures of clonal immunohistochemical loss of MMR expression in tumor cells: clonal loss of MLH1 and PMS2 expression in approximately half of the tumor cells.

Table K. Classification of clonal microsatellite status for case 5.

Clone proportion	Site	μ	σ	π	Normal	MS	MSIsensor	MSIsensor-pro	MSINGS
[0.671, 0.329]	BAT26	24.62<br>22.3	1.5<br>3.31	0.671<br>0.329	24.10<br>2.21	MSI<br>MSI	MSS	MSS	MSS
	NR24	22.57<br>19.85	1.2<br>1.91	0.671<br>0.329	21.85<br>1.95	MSI<br>MSI	MSS	MSS	MSS
	BAT25	25.11<br>22.43	1.23<br>1.83	0.671<br>0.329	24.31<br>2.19	MSI<br>MSI	MSS	MSS	MSS
	NR27	22.04<br>24.3	2.3<br>1.32	0.329<br>0.671	23.58<br>1.96	MSI<br>MSI	MSS	MSS	MSS
	NR21	22.32<br>21.05	1.66<br>2.75	0.671<br>0.329	22.13<br>2.00	MSS<br>MSI	MSS	MSS	MSI
	MS1	13.89<br>12.96	0.52<br>1.45	0.671<br>0.329	13.63<br>0.97	MSI<br>MSI	MSS	MSS	MSS
	MS4	13.28<br>13.31	1.09<br>0.63	0.671<br>0.329	13.41<br>0.84	MSI<br>MSI	MSS	MSS	MSS
	MS5	16.92<br>17.48	1.24<br>0.6	0.671<br>0.329	16.98<br>1.23	MSS<br>MSI	MSS	MSS	MSS
	MS8	15.81	0.96	1	15.81<br>1.00	MSS	MSS	MSS	MSS
	MS9	15.59	1.16	1	15.70<br>1.19	MSS	MSS	MSS	MSS
	MS10	14.71	1.03	1	14.71<br>1.12	MSS	MSS	MSS	MSS
	MS12	15.27<br>14.0	0.99<br>1.8	0.671<br>0.329	14.81<br>1.29	MSI<br>MSI	MSS	MSS	MSS
	MS15	17.91	1.19	1	18.01<br>1.30	MSS	MSS	MSS	MSS
	MS22	16.43	1.57	1	16.48<br>1.45	MSS	MSS	MSS	MSS
	MS23	17.12	1.46	1	17.28<br>1.43	MSS	MSS	MSS	MSS
Sample							MSS	MSS	MSS
MANTIS							MSS		
IHC							partial MSI		

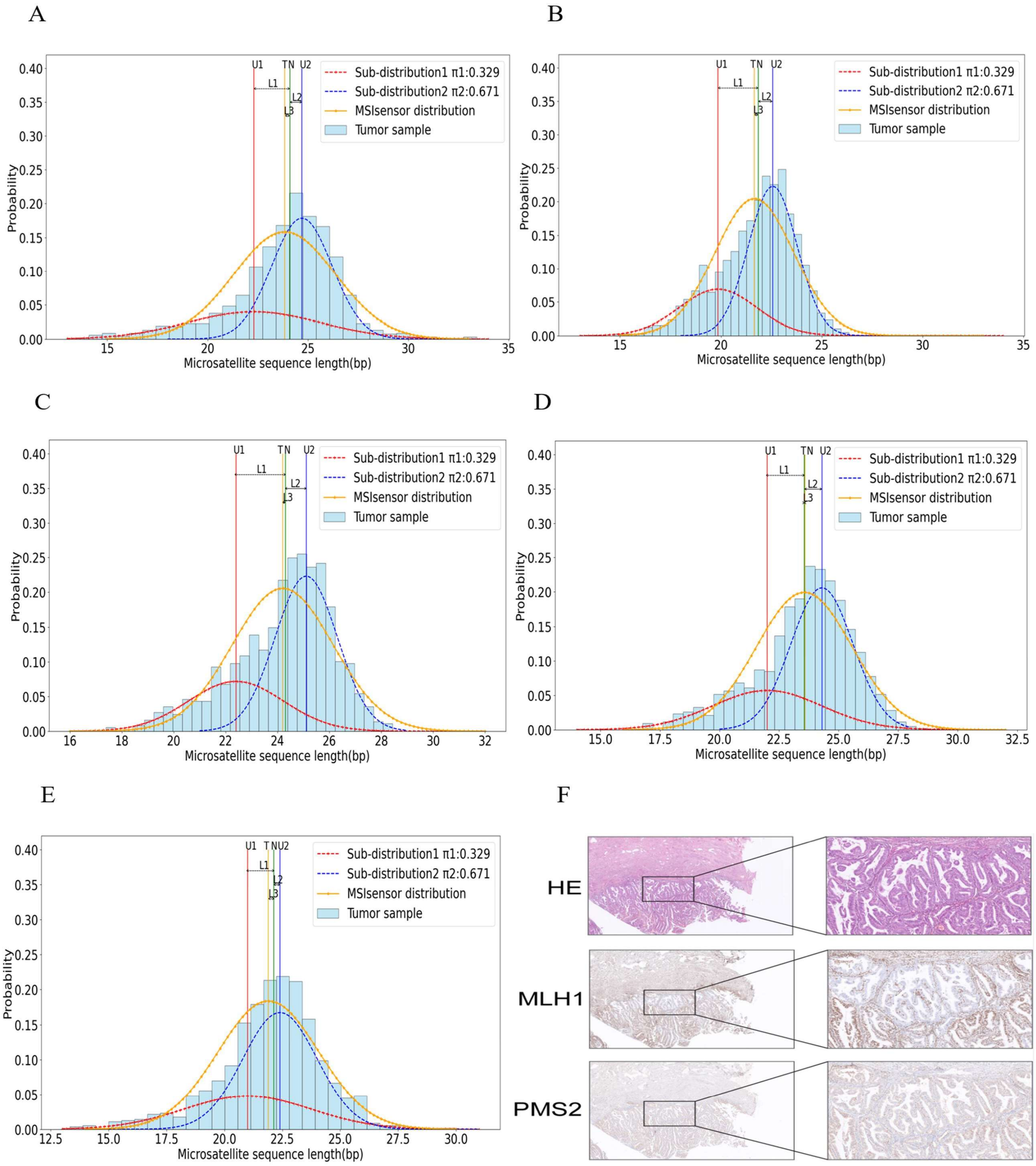

Figure C. IHC detection map and PCR microsatellite detection site length distribution map of case 5. **(A)** BAT26 microsatellite length distribution map **(B)** NR24 microsatellite length distribution map **(C)** BAT25 microsatellite length distribution map **(D)** NR27 microsatellite length distribution map **(E)** NR21 microsatellite length distribution map. **(F)** The figures of clonal immunohistochemical loss of MMR expression in tumor cells: clonal loss of MLH1 and PMS2 protein in focal tumor cells.

Table L. Classification of clonal microsatellite status for case 6.

Clone proportion	Site	μ	σ	π	Normal	MS	MSIsensor	MSIsensor-pro	MSINGS
[0.08, 0.69, 0.15, 0.08]	BAT26	24.44<br>21.57	1.58<br>3.4	0.69<br>0.31	23.75<br>2.65	MSI<br>MSI	MSS	MSS	MSS
	NR24	21.06<br>22.35	2.02<br>0.9	0.69<br>0.31	21.50<br>1.76	MSI<br>MSI	MSS	MSS	MSS
	BAT25	23.28<br>25.27	2.21<br>1.31	0.31<br>0.69	24.62<br>1.79	MSI<br>MSI	MSS	MSS	MSS
	NR27	21.22<br>23.84	2.38<br>1.31	0.31<br>0.69	23.30<br>2.02	MSI<br>MSI	MSS	MSS	MSS
	NR21	21.85<br>22.78	1.96<br>0.75	0.69<br>0.31	22.20<br>1.76	MSI<br>MSI	MSS	MSS	MSS
	MS1	13.9,<br>12.78,	0.59<br>1.38	0.69<br>0.31	13.58<br>1.04	MSI<br>MSI	MSS	MSS	MSS
	MS4	12.94	0.75	1	12.97<br>0.71	MSS	MSS	MSS	MSS
	MS5	17.02	1.26	1	17.09<br>1.21	MSS	MSS	MSS	MSS
	MS8	15.83	1.04	1	15.83<br>0.96	MSS	MSS	MSS	MSS
	MS9	15.92<br>16.41	1.6<br>0.58	0.69<br>0.31	16.16<br>1.23	MSI<br>MSI	MSS	MSS	MSS
	MS10	14.94<br>13.75	0.63<br>1.79	0.84<br>0.16	14.71<br>1.06	MSI<br>MSI	MSS	MSS	MSS
	MS12	12.89<br>15.21	1.08<br>1.03	0.15<br>0.85	14.88<br>1.29	MSI<br>MSI	MSS	MSS	MSS
	MS15	18.04	1.03	1	18.04<br>1.23	MSS	MSS	MSS	MSS
	MS22	15.62	1.18	1	15.74<br>1.09	MSS	MSS	MSS	MSS
	MS23	16.76	1.36	1	16.82<br>1.27	MSS	MSS	MSS	MSS
Sample							MSS	MSS	MSS
MANTIS							MSS		
IHC							partial MSI		

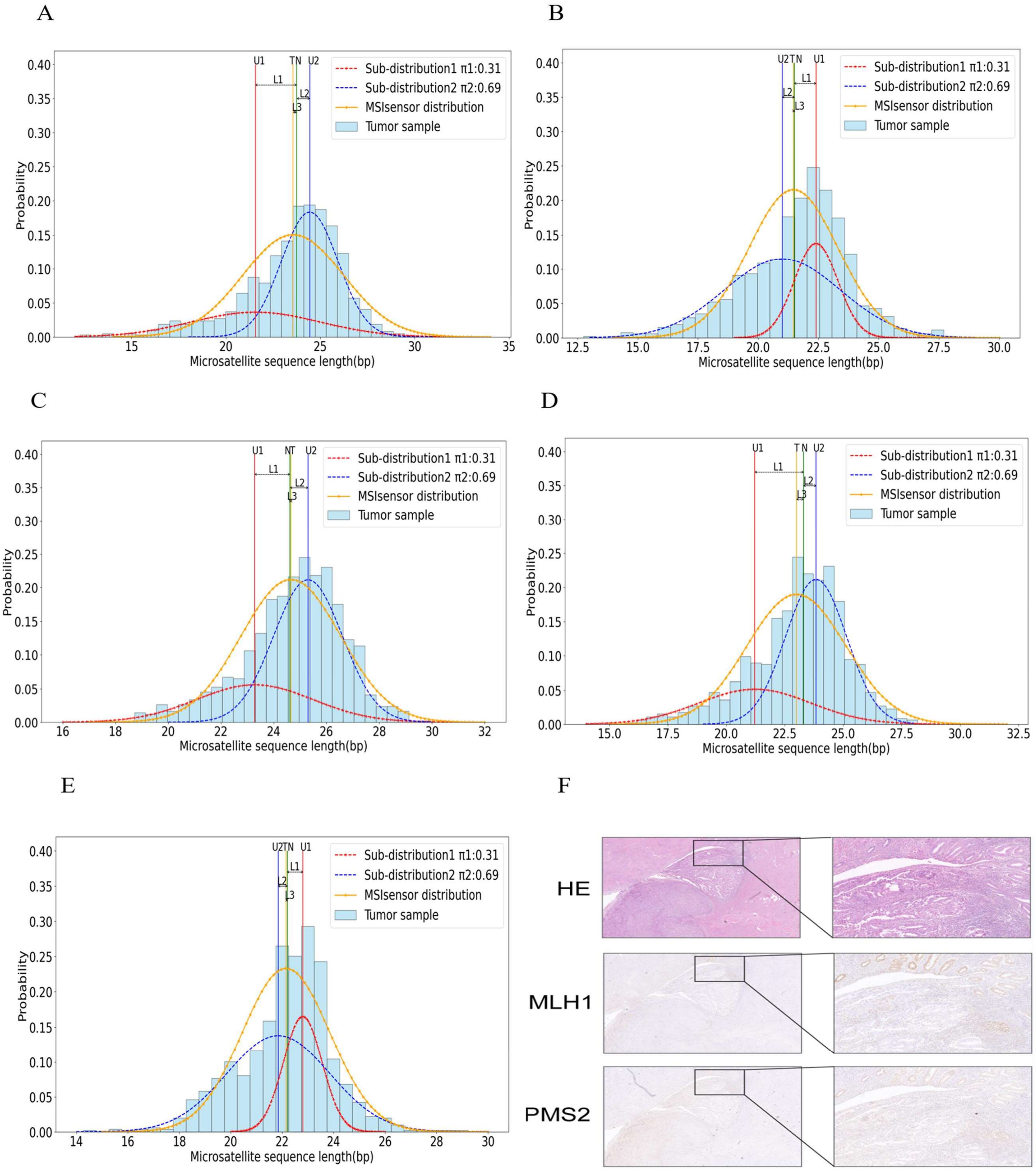

Figure D. IHC detection map and PCR microsatellite detection site length distribution map of case 6. **(A)** BAT26 microsatellite length distribution map **(B)** NR24 microsatellite length distribution map **(C)** BAT25 microsatellite length distribution map **(D)** NR27 microsatellite length distribution map **(E)** NR21 microsatellite length distribution map. **(F)** The figures of clonal immunohistochemical loss of MMR expression in tumor cells: clonal loss of MLH1 and PMS2 protein in the majority of tumor cells.

Table M. Classification of clonal microsatellite status for case 7.

Clone proportion	Site	μ	σ	π	Normal	MS	MSIsensor	MSIsensor-pro	MSINGS
[0.66,<br>0.124,<br>0.132,<br>0.084]	BAT26	23.10<br>24.08<br>22.18	1.37<br>1.08<br>2.67	0.124<br>0.216<br>0.66	22.81<br>2.24	MSS<br>MSI<br>MSI	MSS	MSS	MSS
	NR24	22.07	1.78	1	21.97<br>1.87	MSS	MSS	MSS	MSS
	BAT25	24.1<br>21.32	1.27<br>1.76	0.792<br>0.208	23.61<br>1.89	MSI<br>MSI	MSS	MSS	MSS
	NR27	21.04<br>23.98	2.12<br>1.31	0.34<br>0.66	23.12<br>1.93	MSI<br>MSI	MSS	MSS	MSS
	NR21	22.16	3.45	1	22.08<br>1.85	MSS	MSS	MSS	MSS
	MS1	13.95<br>12.93	0.52<br>1.44	0.66<br>0.34	13.63<br>1.07	MSI<br>MSI	MSS	MSS	MSS
	MS4	12.89	0.71	1	12.89<br>0.79	MSS	MSS	MSS	MSS
	MS5	16.92<br>17.79	1.6<br>0.7	0.34<br>0.66	17.57<br>1.16	MSI<br>MSI	MSS	MSS	MSS
	MS8	15.83	0.99	1	15.83<br>1.05	MSS	MSS	MSS	MSS
	MS9	16.06<br>16.62	1.5<br>0.57	0.66<br>0.34	16.15<br>1.36	MSS<br>MSI	MSS	MSS	MSS
	MS10	14.73	1.07	1	14.74<br>1.03	MSS	MSS	MSS	MSS
	MS12	13.66<br>14.8	1.57<br>0.63	0.34<br>0.66	14.45<br>1.18	MSI<br>MSI	MSS	MSS	MSS
	MS15	18.01	1.34	1	18.15<br>1.05	MSS	MSS	MSS	MSS
	MS22	15.72	1.18	1	15.77<br>1.01	MSS	MSS	MSS	MSS
		17.89	1.85	0.66	18.30	MSI			
	MS23	18.59	0.72	0.34	1.47	MSI	MSS	MSS	MSS
Sample							MSS	MSS	MSS
MANTIS								MSS	
IHC								partial MSI	

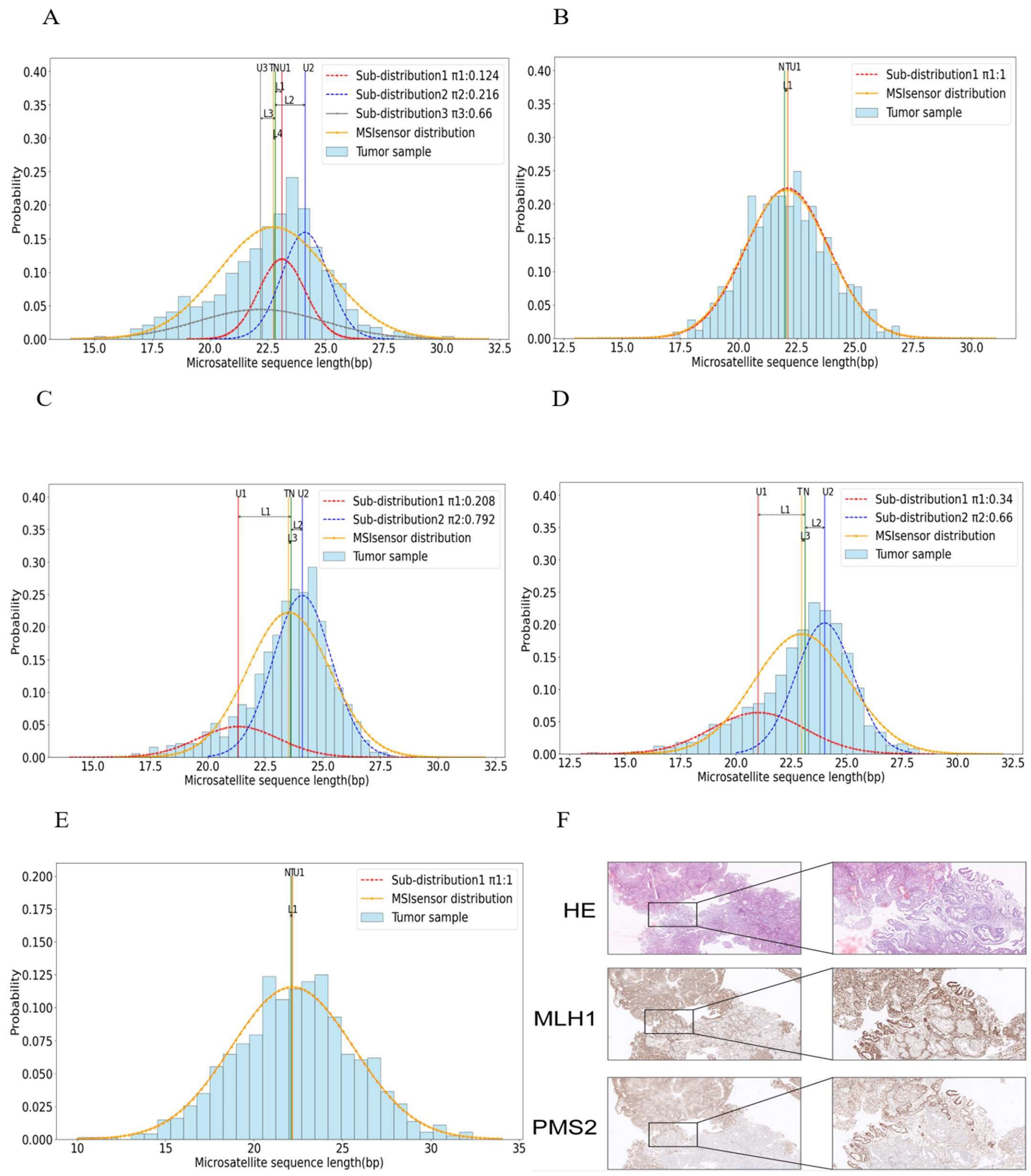

Figure E. IHC detection map and PCR microsatellite detection site length distribution map of case 7. **(A)** BAT26 microsatellite length distribution map **(B)** NR24 microsatellite length distribution map **(C)** BAT25 microsatellite length distribution map **(D)** NR27 microsatellite length distribution map **(E)** NR21 microsatellite length distribution map. **(F)** The figures of clonal immunohistochemical loss of MMR expression in tumor cells: clonal loss of MLH1 and PMS2 protein in approximately half of the tumor cells.

Table N. Classification of clonal microsatellite status for case 8.

Clone proportion	Site	μ	σ	π	Normal	MS	MSIsensor	MSIsensor-pro	MSINGS
[0.63, 0.21, 0.16]	BAT26	25.38<br>23.60<br>20.78	0.95<br>1.75<br>3.01	0.21<br>0.63<br>0.16	23.59<br>2.65	MSI<br>MSS<br>MSI	MSS	MSS	MSS
	NR24	21.52<br>22.77	2.24<br>0.82	0.63<br>0.37	21.93<br>1.97	MSI<br>MSI	MSS	MSS	MSS
	BAT25	25.50<br>21.66<br>24.34	1.06<br>1.89<br>1.14	0.21<br>0.16<br>0.63	24.15<br>1.95	MSI<br>MSI<br>MSS	MSS	MSS	MSS
	NR27	23.02<br>21.00<br>24.40	1.61<br>2.78<br>0.88	0.63<br>0.16<br>0.21	23.16<br>1.87	MSS<br>MSI<br>MSI	MSS	MSS	MSS
	NR21	23.05<br>22.22<br>20.16	0.65<br>1.57<br>2.43	0.21<br>0.63<br>0.16	22.08<br>1.78	MSI<br>MSS<br>MSI	MSS	MSS	MSS
	MS1	13.8<br>14.46	1.46<br>0.66	0.63<br>0.37	14.08<br>1.23	MSI<br>MSI	MSS	MSS	MSI
	MS4	13.05<br>11.86	0.59<br>0.61	0.84<br>0.16	12.96<br>0.74	MSS<br>MSI	MSS	MSS	MSS
	MS5	17.2<br>16.88	0.95<br>1.91	0.84<br>0.16	17.18<br>1.20	MSS<br>MSI	MSS	MSS	MSS
	MS8	15.75	1.00	1	15.85<br>1.00	MSI	MSS	MSS	MSS
	MS9	16.93<br>15.84<br>13.94	0.69<br>0.54<br>1.33	0.16<br>0.63<br>0.21	15.71<br>1.12	MSI<br>MSS<br>MSI	MSS	MSS	MSS
	MS10	13.06<br>13.87	1.55<br>0.66	0.16<br>0.84	13.81<br>0.92	MSI<br>MSS	MSS	MSS	MSS
	MS12	13.81<br>14.85	1.54<br>0.59	0.37<br>0.63	14.50<br>1.14	MSI<br>MSI	MSS	MSS	MSS
	MS15	18.20	1.45	1	18.06<br>1.13	MSS	MSS	MSS	MSS
	MS22	16.64<br>15.89	1.4<br>1.96	0.79<br>0.21	16.48<br>1.57	MSS<br>MSI	MSS	MSS	MSS
	MS23	18.64	1.44	1	18.80<br>1.44	MSS	MSS	MSS	MSS
Sample							MSS	MSS	MSS
MANTIS								MSS	
IHC								partial MSI	

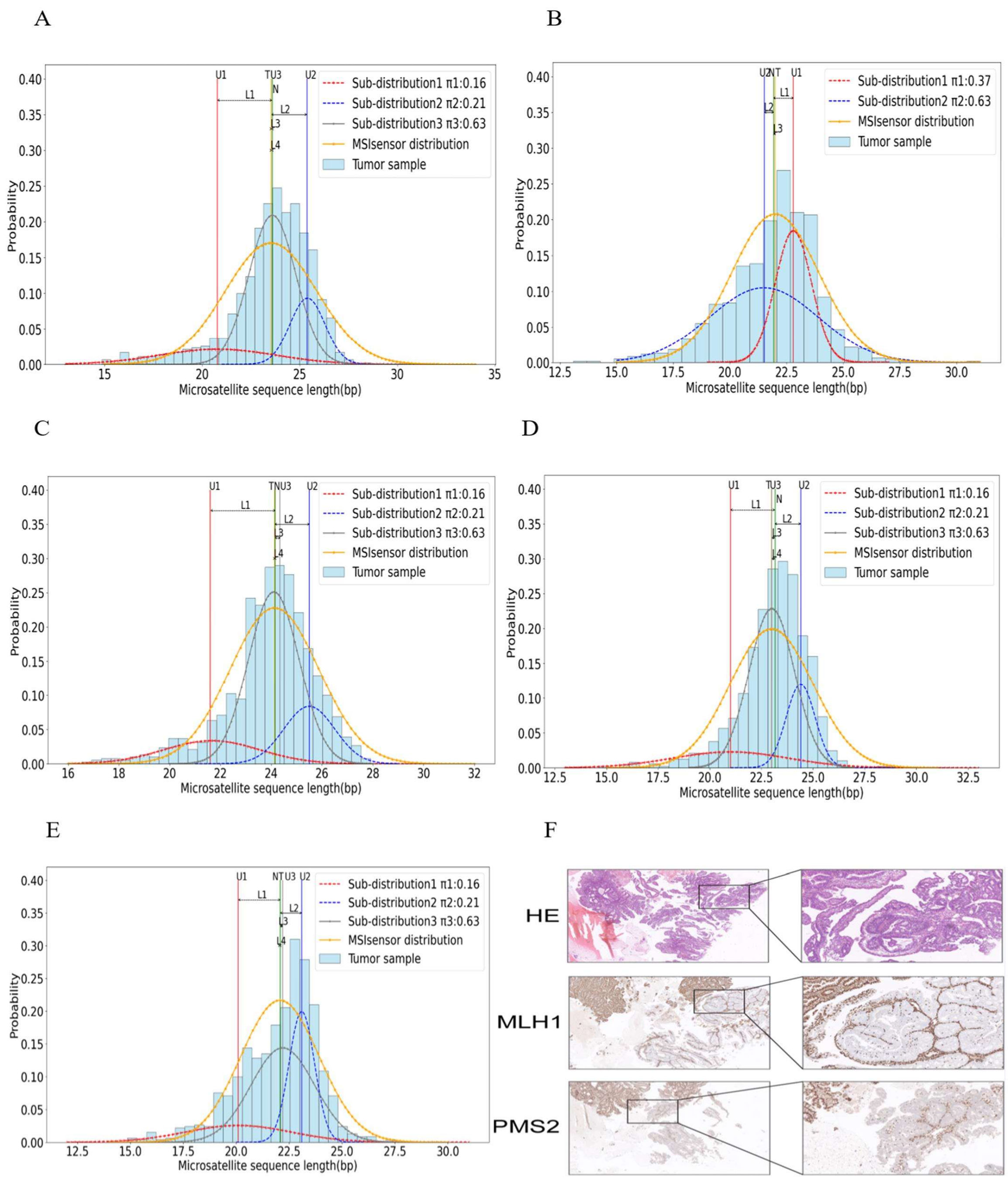

Figure F. IHC detection map and PCR microsatellite detection site length distribution map of case 8. **(A)** BAT26 microsatellite length distribution map **(B)** NR24 microsatellite length distribution map **(C)** BAT25 microsatellite length distribution map **(D)** NR27 microsatellite length distribution map **(E)** NR21 microsatellite length distribution map. **(F)** The figures of clonal immunohistochemical loss of MMR expression in tumor cells: clonal loss of MLH1 and PMS2 protein in approximately half of the tumor cells.

Table O. Classification of clonal microsatellite status for case 9.

Clone proportion	Site	μ	σ	π	Normal	MS	MSIsensor	MSIsensor-pro	MSINGS
[0.04, 0.11, 0.75, 0.1]		20.03	3.67	0.25	16.94	MSI			
	BAT26	12.52	1.13	0.75	5.72	MSI	MSI	MSI	MSS
		20.32	2.21	0.75	21.23	MSI			
	NR24	15.97	1.57	0.25	2.16	MSI	MSI	MSI	MSS
		20.89	2.70	0.75	24.16	MSI			
	BAT25	23.99	1.75	0.25	2.41	MSS	MSI	MSI	MSI
		19.12	2.14	0.75	23.03	MSI			
	NR27	24.10	1.64	0.25	2.53	MSI	MSI	MSI	MSI
		17.55	2.02	0.75	21.61	MSI			
	NR21	22.21	1.38	0.25	2.38	MSI	MSI	MSI	MSI
		12.41	1.15	0.79	13.51	MSI			
	MS1	13.66	0.73	0.21	1.14	MSI	MSI	MSI	MSI
		12.66	0.79	0.79	12.83	MSI			
	MS4	11.0	0.59	0.21	0.81	MSI	MSI	MSI	MSI
		17.99	0.57	0.25	17.41	MSI			
	MS5	16.16	1.51	0.75	1.21	MSI	MSI	MSI	MSI
		15.74	0.92	0.75	15.69	MSS			
	MS8	12.86	0.63	0.25	1.16	MSI	MSI	MSI	MSS
		15.74	0.69	0.25	15.69	MSS			
	MS9	14.9	1.53	0.75	1.11	MSI	MSI	MSI	MSI
		13.72	1.37	0.75	14.68	MSI			
	MS10	14.38	0.65	0.25	1.19	MSI	MSI	MSI	MSI
		14.0	1.19	0.75	14.37	MSI			
	MS12	12.3	1.12	0.25	1.20	MSI	MSI	MSI	MSS
		18.08	1.49	0.21	17.99	MSS			
	MS15	16.8	1.59	0.79	1.75	MSI	MSI	MSI	MSI
		15.09	1.19	0.75	15.63	MSI			
	MS22	12.2	0.84	0.25	1.41	MSI	MSI	MSI	MSS
		18.79	1.26	0.21	17.89	MSI			
	MS23	15.31	1.6	0.79	1.73	MSI	MSI	MSI	MSI
Sample							MSI	MSI	MSI
MANTIS							MSI		
IHC							partial MSI		

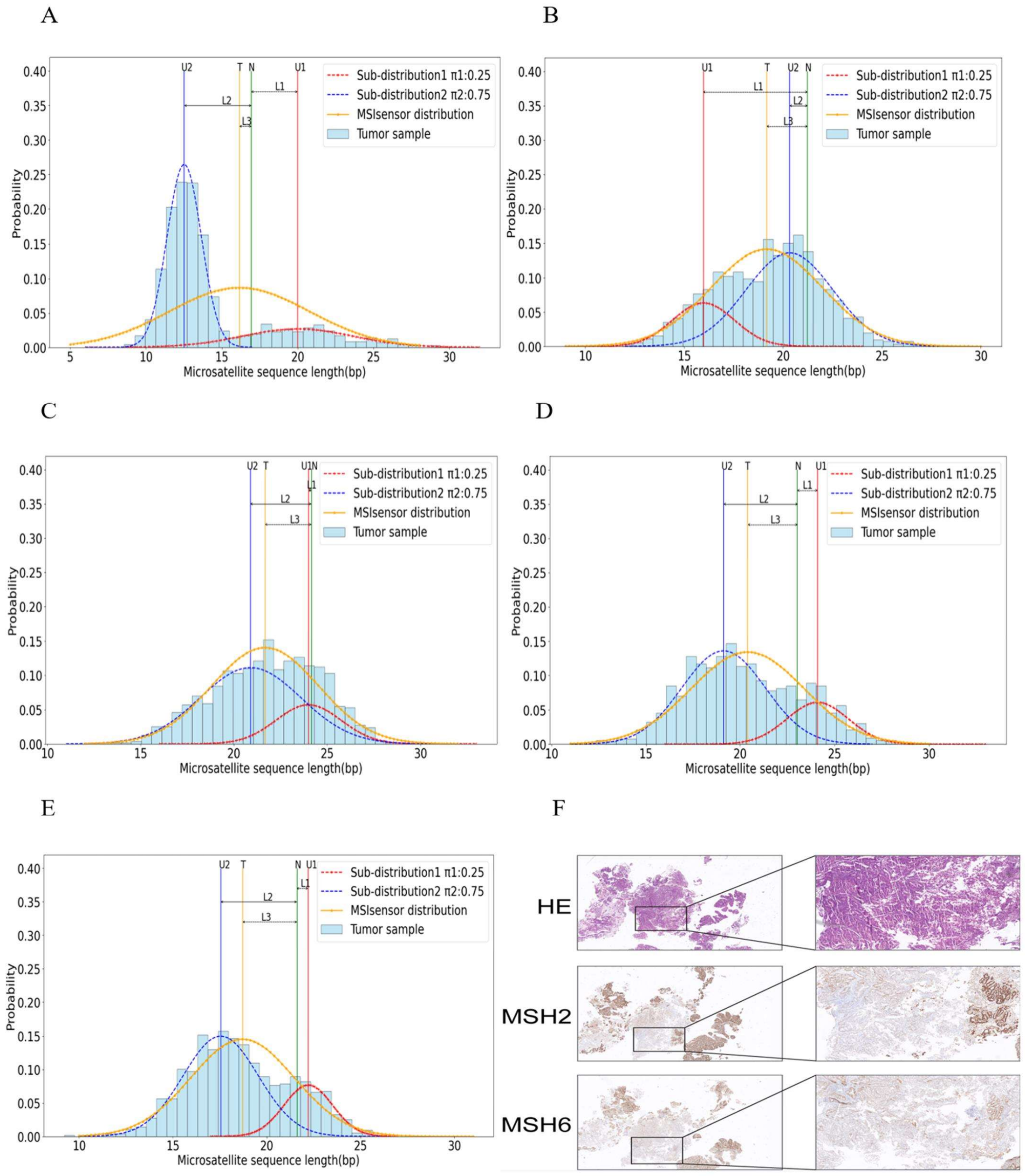

Figure G. IHC detection map and PCR microsatellite detection site length distribution map of case 9. **(A)** BAT26 microsatellite length distribution map **(B)** NR24 microsatellite length distribution map **(C)** BAT25 microsatellite length distribution map **(D)** NR27 microsatellite length distribution map **(E)** NR21 microsatellite length distribution map. **(F)** The figures of clonal immunohistochemical loss of MMR expression in tumor cells: clonal loss of MSH2 and MSH6 protein in the majority of tumor cells.

Table P. Classification of clonal microsatellite status for case 10.

Clone proportion	Site	μ	σ	π	Normal	MS	MSIsensor	MSIsensor-pro	MSINGS
[0.69, 0.08, 0.084, 0.085, 0.061]	BAT26	22.44<br>24.17	2.52<br>1.37	0.69<br>0.31	22.20<br>2.36	MSI<br>MSI	MSI	MSI	MSS
	NR24	21.94<br>20.45	1.33<br>2.48	0.69<br>0.31	22.06<br>1.70	MSS<br>MSI	MSI	MSI	MSS
	BAT25	23.40<br>24.68	2.1<br>0.85	0.69<br>0.31	24.00<br>1.84	MSI<br>MSI	MSS	MSS	MSS
	NR27	23.66<br>21.37	1.65<br>2.37	0.69<br>0.31	23.72<br>2.03	MSS<br>MSI	MSI	MSI	MSS
	NR21	22.19<br>20.78	1.34<br>2.26	0.69<br>0.31	22.33<br>1.67	MSS<br>MSI	MSI	MSI	MSS
	MS1	11.98<br>13.69<br>14.53	0.80<br>0.63<br>0.84	0.169<br>0.69<br>0.141	13.61<br>1.06	MSI<br>MSS<br>MSI	MSI	MSI	MSS
	MS4	12.71<br>12.49	1.49<br>0.77	0.061<br>0.939	12.46<br>0.80	MSI<br>MSS	MSS	MSS	MSS
	MS5	17.82<br>15.89	0.76<br>0.93	0.775<br>0.225	17.62<br>1.09	MSI<br>MSI	MSI	MSI	MSS
	MS8	15.83<br>14.03<br>17.21	0.55<br>1.04<br>0.64	0.751<br>0.165<br>0.084	15.81<br>0.94	MSS<br>MSI<br>MSI	MSS	MSS	MSS
	MS9	15.56	1.12	1	15.75<br>1.07	MSI	MSS	MSS	MSS
	MS10	14.44	1.17	1	14.66<br>1.01	MSI	MSI	MSI	MSS
	MS12	12.19<br>14.74<br>15.24	1.08<br>1.25<br>0.65	0.061<br>0.69<br>0.249	14.83<br>1.30	MSI<br>MSS<br>MSI	MSS	MSS	MSS
	MS15	17.94	1.21	1	18.03<br>1.04	MSS	MSS	MSS	MSS
	MS22	16.49	1.51	1	16.67<br>1.48	MSS	MSS	MSS	MSS
	MS23	18.34<br>16.9	1.2<br>2.04	0.69<br>0.31	18.06<br>1.62	MSS<br>MSI	MSS	MSS	MSS
Sample							MSI	MSI	MSS
MANTIS								MSS	
IHC								partial MSI	

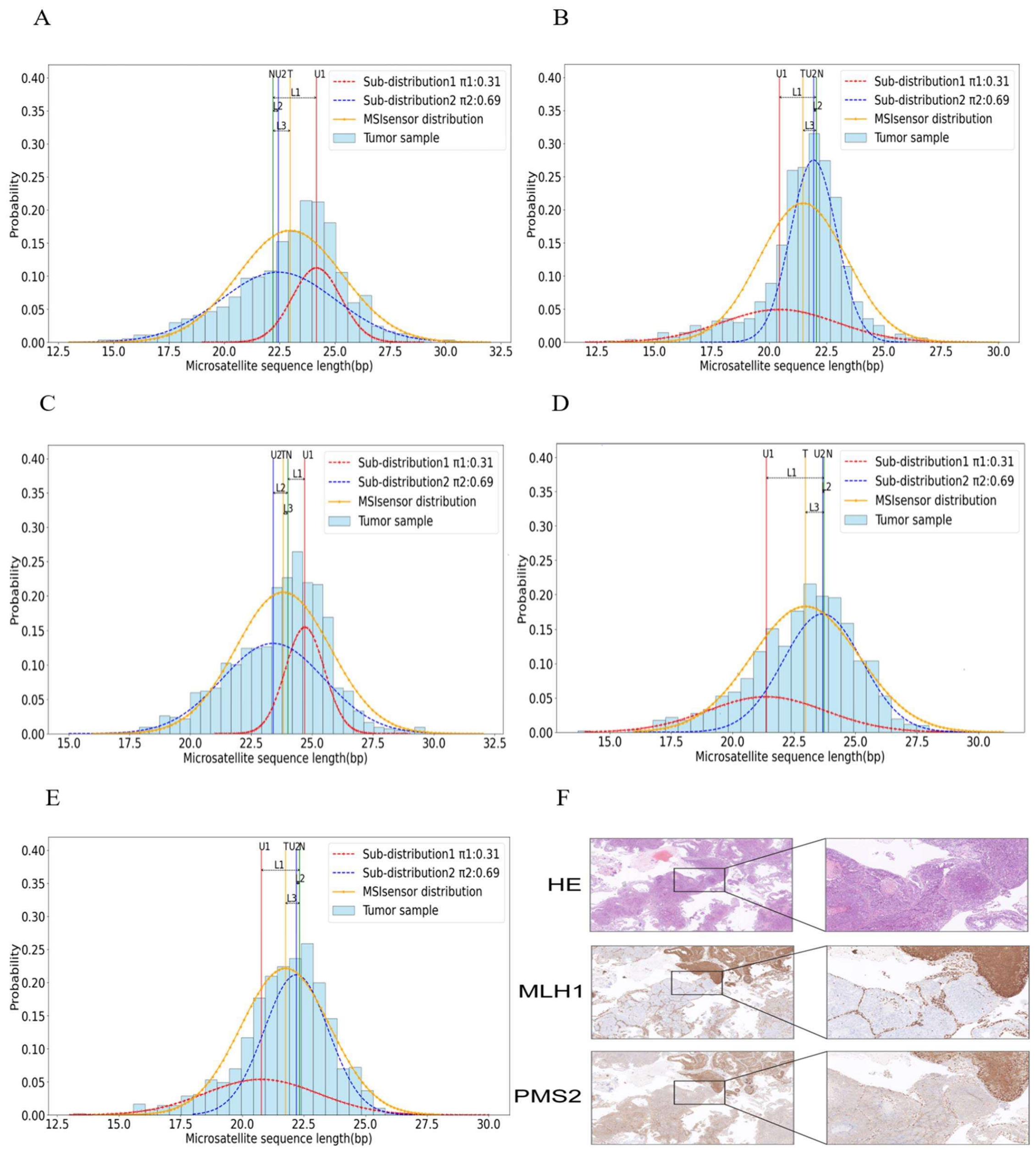

Figure H. IHC detection map and PCR microsatellite detection site length distribution map of case 10. **(A)** BAT26 microsatellite length distribution map **(B)** NR24 microsatellite length distribution map **(C)** BAT25 microsatellite length distribution map **(D)** NR27 microsatellite length distribution map **(E)** NR21 microsatellite length distribution map. **(F)** The figures of clonal immunohistochemical loss of MMR expression in tumor cells: clonal loss of MLH1 and PMS2 protein in the majority of tumor cells.

Table Q. Classification of clonal microsatellite status for case 11.

Clone proportion	Site	μ	σ	π	Normal	MS	MSIsensor	MSIsensor-pro	MSINGS
[0.713, 0.102, 0.095, 0.09]	BAT26	23.89<br>19.40	1.94<br>2.03	0.713<br>0.287	23.76<br>2.12	MSS<br>MSI	MSI	MSI	MSS
	NR24	20.89<br>22.37	2.08<br>1.09	0.713<br>0.287	21.84<br>2.02	MSI<br>MSI	MSI	MSI	MSS
	BAT25	23.14<br>24.5	2.31<br>1.01	0.713<br>0.287	24.04<br>2.20	MSI<br>MSI	MSI	MSI	MSI
	NR27	19.63<br>22.69	1.97<br>1.69	0.287<br>0.713	23.16<br>2.01	MSI<br>MSI	MSI	MSI	MSI
	NR21	21.56<br>18.8	1.51<br>1.96	0.713<br>0.287	21.69<br>1.85	MSS<br>MSI	MSI	MSI	MSS
	MS1	11.66<br>13.8	0.76<br>0.78	0.185<br>0.815	13.67<br>1.05	MSI<br>MSS	MSI	MSI	MSS
	MS4	12.88<br>12.41	0.55<br>1.12	0.713<br>0.287	12.93<br>0.77	MSS<br>MSI	MSI	MSI	MSS
	MS5	17.65<br>15.88	0.96<br>1.14	0.713<br>0.287	17.60<br>1.09	MSS<br>MSI	MSI	MSI	MSS
	MS8	16.43<br>14.01	0.95<br>1.09	0.815<br>0.185	16.36<br>1.15	MSS<br>MSI	MSI	MSI	MSS
	MS9	14.42<br>15.68	0.93<br>1.19	0.185<br>0.815	15.64<br>1.11	MSI<br>MSS	MSI	MSI	MSI
	MS10	14.71<br>12.89	0.73<br>0.94	0.713<br>0.287	14.74<br>1.00	MSS<br>MSI	MSI	MSI	MSI
	MS12	14.77<br>12.46	1.31<br>1.06	0.713<br>0.287	14.86<br>1.30	MSS<br>MSI	MSI	MSI	MSI
	MS15	17.96<br>15.70	1.05<br>1.07	0.713<br>0.287	18.08<br>1.37	MSS<br>MSI	MSI	MSI	MSI
	MS22	14.44<br>16.66	1.17<br>1.53	0.287<br>0.713	16.50<br>1.59	MSI<br>MSS	MSI	MSI	MSS
	MS23	16.42	1.41	1	16.90<br>1.18	MSI	MSI	MSI	MSS
Sample							MSI	MSI	MSI
MANTIS								MSS	
IHC								partial MSI	

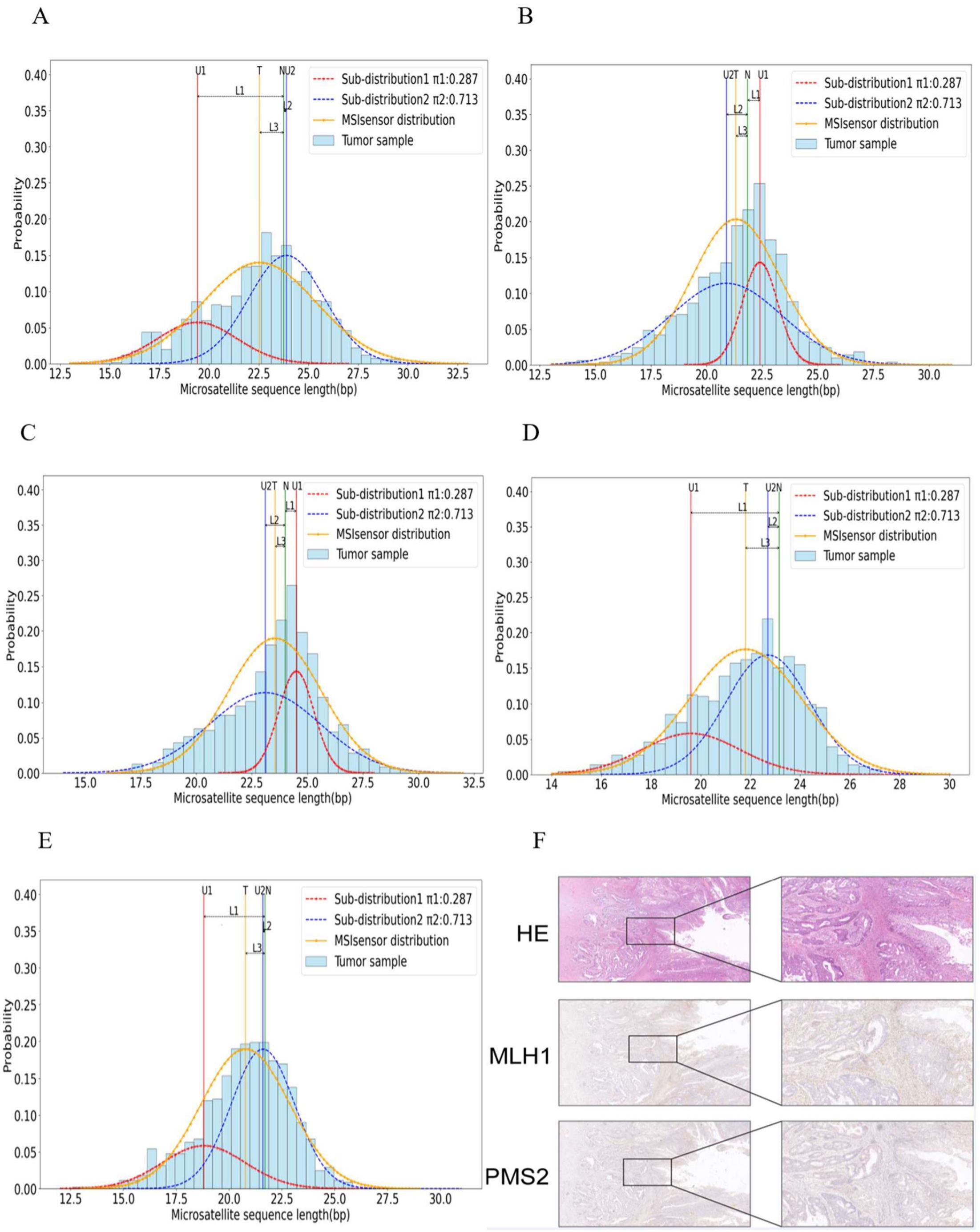

Figure I. IHC detection map and PCR microsatellite detection site length distribution map of case 11. **(A)** BAT26 microsatellite length distribution map **(B)** NR24 microsatellite length distribution map **(C)** BAT25 microsatellite length distribution map **(D)** NR27 microsatellite length distribution map **(E)** NR21 microsatellite length distribution map. **(F)** The figures of clonal immunohistochemical loss of MMR expression in tumor cells: clonal loss of MLH1 and PMS2 protein in the majority of tumor cells.

Table R. Classification of clonal microsatellite status for case 12.

Clone proportion	Site	μ	σ	π	Normal	MS	MSIsensor	MSIsensor-pro	MSINGS
[0.7, 0.3]	BAT26	24.31<br>23.04	1.83<br>4.29	0.7<br>0.3	23.86<br>2.29	MSI<br>MSI	MSS	MSS	MSS
	NR24	20.13<br>22.45	2.54<br>1.46	0.3<br>0.7	22.14<br>1.78	MSI<br>MSI	MSI	MSI	MSS
	BAT25	24.52<br>21.35	1.38<br>2.16	0.7<br>0.3	24.20<br>1.78	MSI<br>MSI	MSI	MSI	MSS
	NR27	23.74<br>25.14	1.97<br>0.97	0.7<br>0.3	24.15<br>2.02	MSI<br>MSI	MSI	MSS	MSS
	NR21	22.2<br>21.54	1.44<br>2.26	0.7<br>0.3	22.24<br>1.81	MSS<br>MSI	MSI	MSS	MSS
	MS1	14.02<br>13.03	0.51<br>1.31	0.7<br>0.3	13.66<br>1.02	MSI<br>MSI	MSS	MSS	MSS
	MS4	12.88	0.76	1	12.96<br>0.78	MSI	MSS	MSS	MSS
	MS5	17.56	1.09	1	17.61<br>1.10	MSS	MSS	MSS	MSS
	MS8	15.96<br>17.52	1.4<br>0.87	0.3<br>0.7	17.22<br>1.17	MSI<br>MSI	MSS	MSS	MSS
	MS9	14.63<br>16.02	1.41<br>0.54	0.3<br>0.7	15.76<br>1.09	MSI<br>MSI	MSS	MSS	MSS
	MS10	14.99<br>14.26	0.57<br>1.36	0.7<br>0.3	14.75<br>1.03	MSI<br>MSI	MSS	MSS	MSS
	MS12	13.61<br>15.18	1.35<br>1.00	0.3<br>0.7	14.89<br>1.31	MSI<br>MSI	MSS	MSS	MSS
	MS15	18.14	1.11	1	18.07<br>1.21	MSS	MSS	MSS	MSS
	MS22	16.50	1.53	1	16.57<br>1.45	MSS	MSS	MSS	MSS
	MS23	17.47	1.53	1	17.77<br>1.28	MSI	MSI	MSI	MSS
Sample							MSI	MSI	MSS
MANTIS								MSS	
IHC								partial MSI	

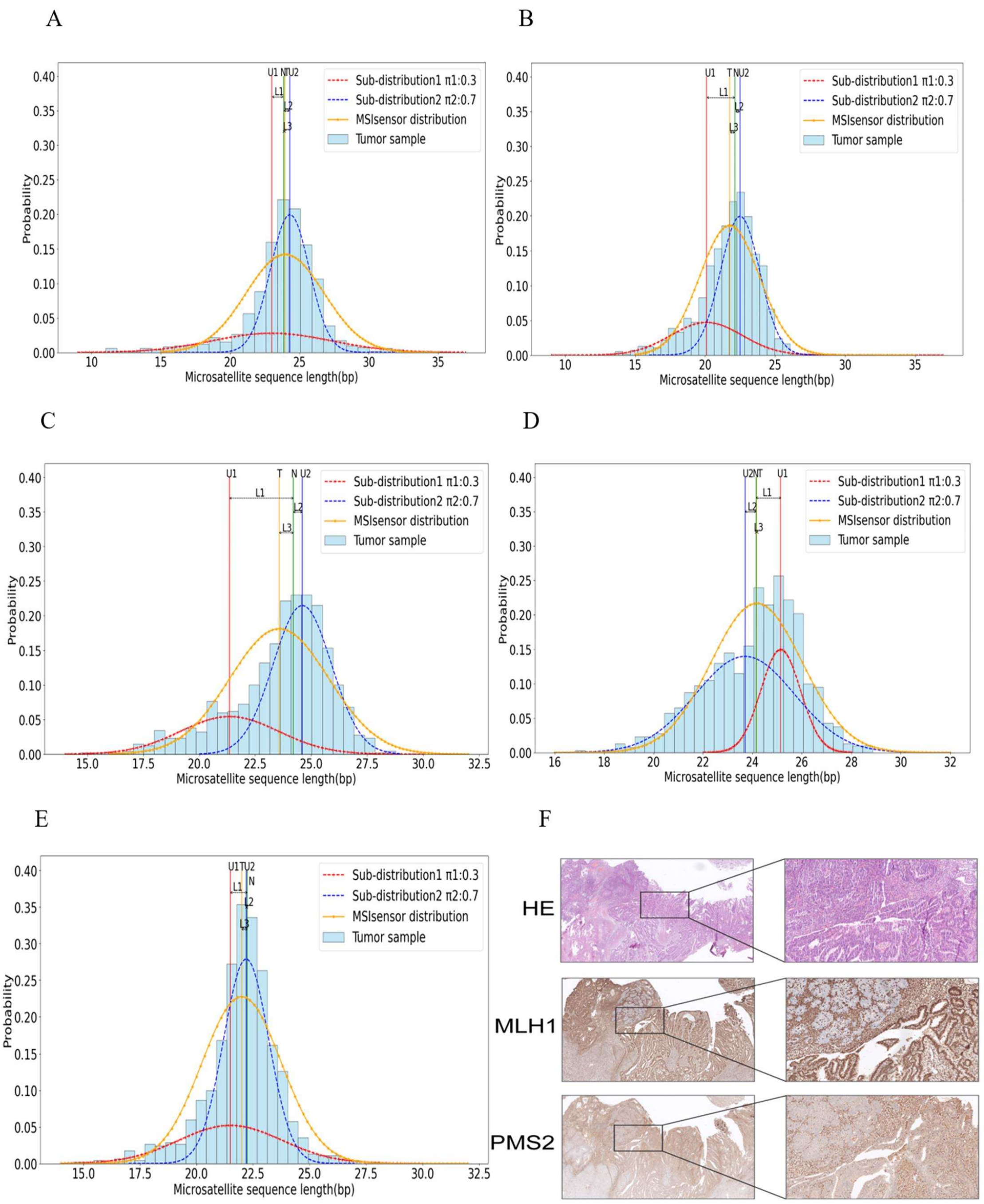

Figure J. IHC detection map and PCR microsatellite detection site length distribution map of case 12. **(A)** BAT26 microsatellite length distribution map **(B)** NR24 microsatellite length distribution map **(C)** BAT25 microsatellite length distribution map **(D)** NR27 microsatellite length distribution map **(E)** NR21 microsatellite length distribution map. **(F)** The figures of clonal immunohistochemical loss of MMR expression in tumor cells: clonal loss of MLH1 and PMS2 protein in focal tumor cells.

Table S. Classification of clonal microsatellite status for case 13.

Clone proportion	Site	μ	σ	π	Normal	MS	MSIsensor	MSIsensor-pro	MSINGS
[0.68, 0.17, 0.15]	BAT26	24.35<br>21.75	1.66<br>3.35	0.68<br>0.32	23.94<br>2.42	MSI<br>MSI	MSS	MSS	MSS
	NR24	20.57<br>22.07	2.56<br>1.18	0.32<br>0.68	21.62<br>1.90	MSI<br>MSI	MSS	MSS	MSS
	BAT25	24.37<br>21.46<br>25.05	1.27<br>2.21<br>1.28	0.68<br>0.15<br>0.17	24.06<br>1.99	MSS<br>MSI<br>MSI	MSS	MSS	MSS
	NR27	24.17<br>22.82	0.98<br>2.04	0.32<br>0.68	23.15<br>2.03	MSI<br>MSI	MSS	MSS	MSS
	NR21	22.88<br>21.98	1.25<br>2.61	0.68<br>0.32	22.68<br>1.94	MSS<br>MSI	MSS	MSS	MSS
	MS1	13.80<br>12.22<br>15.06	0.42<br>0.76<br>0.35	0.68<br>0.17<br>0.15	13.71<br>0.95	MSS<br>MSI<br>MSI	MSS	MSS	MSS
	MS4	12.91	0.73	1	12.96<br>0.74	MSS	MSS	MSS	MSS
	MS5	17.64	1.33	1	17.64<br>1.26	MSS	MSS	MSS	MSS
	MS8	16.21<br>16.55	1.37<br>0.62	0.68<br>0.32	16.23<br>1.14	MSS<br>MSI	MSS	MSS	MSS
	MS9	16.34<br>14.00<br>15.89	1.50<br>1.45<br>0.79	0.17<br>0.15<br>0.68	15.71<br>1.24	MSI<br>MSI<br>MSS	MSS	MSS	MSS
	MS10	14.84<br>13.35<br>15.99	0.47<br>1.15<br>0.68	0.68<br>0.17<br>0.15	14.74<br>1.00	MSS<br>MSI<br>MSI	MSS	MSS	MSS
	MS12	14.74<br>12.35	0.86<br>0.98	0.85<br>0.15	14.48<br>1.16	MSI<br>MSI	MSS	MSS	MSS
	MS15	16.71<br>18.06<br>19.29	1.78<br>0.64<br>1.06	0.17<br>0.68<br>0.15	18.16<br>1.13	MSI<br>MSS<br>MSI	MSS	MSS	MSS
	MS22	16.7<br>17.86	1.97<br>0.71	0.32<br>0.68	17.63<br>1.32	MSI<br>MSI	MSS	MSS	MSS
	MS23	17.67	1.53	1	17.77<br>1.39	MSS	MSS	MSS	MSS
Sample							MSS	MSS	MSS
MANTIS								MSS	
IHC								partial MSI	

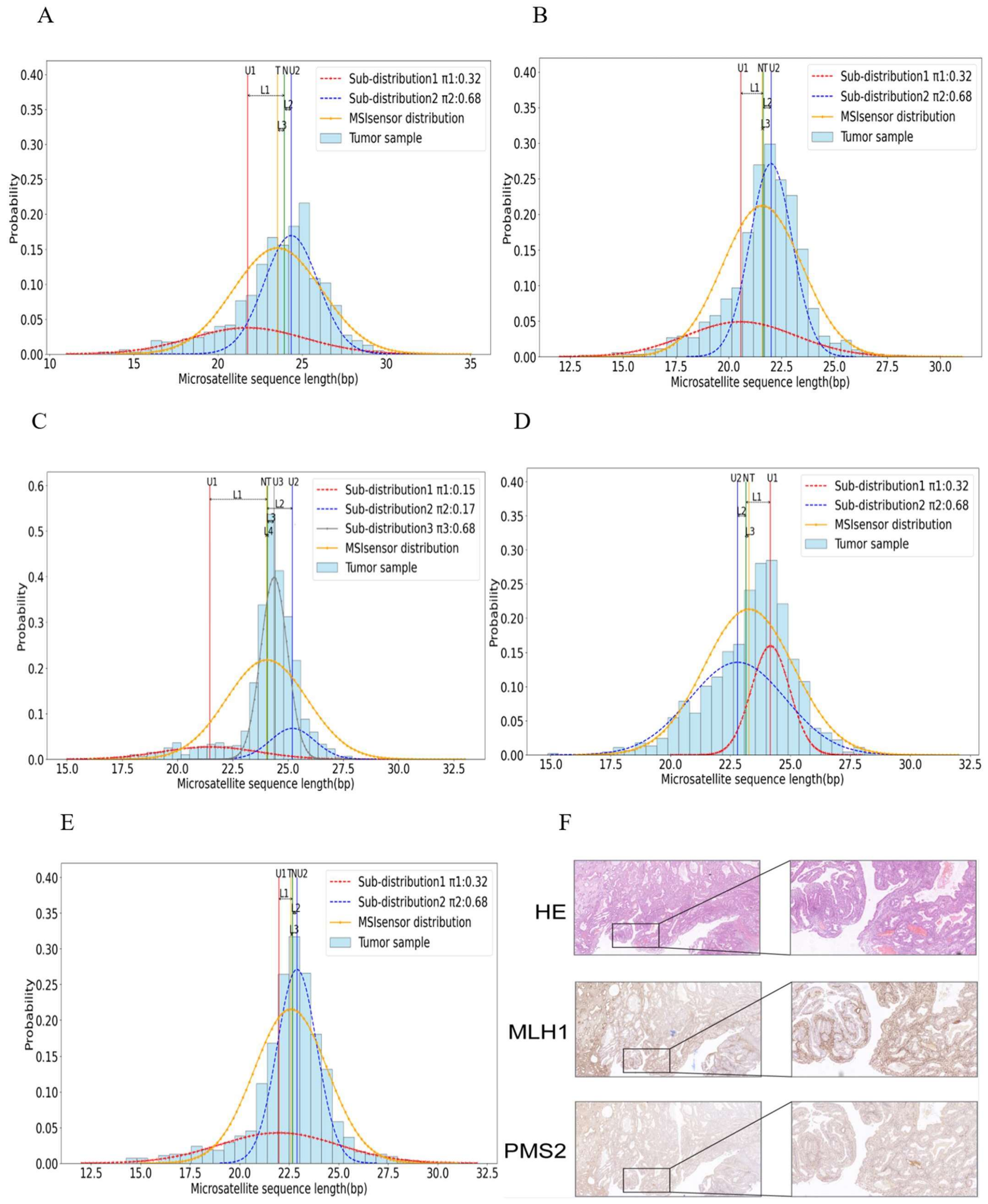

Figure K. IHC detection map and PCR microsatellite detection site length distribution map of case 13. **(A)** BAT26 microsatellite length distribution map **(B)** NR24 microsatellite length distribution map **(C)** BAT25 microsatellite length distribution map **(D)** NR27 microsatellite length distribution map **(E)** NR21 microsatellite length distribution map. **(F)** The figures of clonal immunohistochemical loss of MMR expression in tumor cells: clonal loss of MLH1 and PMS2 protein in the majority of tumor cells.

Table T. Classification of clonal microsatellite status for case 14.

Clone proportion	Site	μ	σ	π	Normal	MS	MSIsensor	MSIsensor-pro	MSINGS
[0.684, 0.142, 0.174]	BAT26	17.73<br>24.30	2.64<br>1.77	0.174<br>0.826	23.69<br>2.35	MSI<br>MSI	MSI	MSI	MSS
	NR24	21.24<br>22.60	2.23<br>0.91	0.684<br>0.316	22.03<br>1.95	MSI<br>MSI	MSS	MSS	MSS
	BAT25	24.74<br>22.67	1.13<br>2.47	0.684<br>0.316	24.10<br>1.75	MSI<br>MSI	MSS	MSS	MSS
	NR27	20.23<br>23.97	2.16<br>1.31	0.316<br>0.684	23.10<br>2.08	MSI<br>MSI	MSS	MSS	MSS
	NR21	19.98<br>22.70	2.79<br>1.13	0.316<br>0.684	22.14<br>1.80	MSI<br>MSI	MSI	MSI	MSS
	MS1	13.81<br>11.68	0.75<br>0.86	0.858<br>0.142	13.61<br>1.07	MSI<br>MSI	MSS	MSS	MSS
	MS4	12.78	0.86	1	12.91<br>0.76	MSI	MSI	MSI	MSS
	MS5	12.78	0.86	1	12.91<br>0.76	MSI	MSI	MSI	MSS
	MS8	15.74	1.04	1	15.80<br>1.02	MSS	MSS	MSS	MSS
	MS9	15.97<br>14.08<br>16.11	0.68<br>1.23<br>1.54	0.684<br>0.174<br>0.142	15.83<br>1.07	MSS<br>MSI<br>MSI	MSS	MSS	MSS
	MS10	14.68	1.11	1	14.74<br>1.01	MSS	MSI	MSI	MSS
	MS12	12.87<br>14.84	2.02<br>0.63	0.316<br>0.684	14.41<br>1.16	MSI<br>MSI	MSI	MSI	MSS
	MS15	17.91	1.22	1	18.11<br>1.15	MSS	MSS	MSS	MSS
	MS22	16.45<br>15.86	0.62<br>1.58	0.316<br>0.684	16.19<br>1.26	MSI<br>MSI	MSS	MSS	MSS
	MS23	17.15<br>15.11	0.74<br>2.29	0.684<br>0.316	16.78<br>1.23	MSI<br>MSI	MSI	MSI	MSS
Sample							MSI	MSI	MSS
MANTIS								MSS	
IHC								partial MSI	

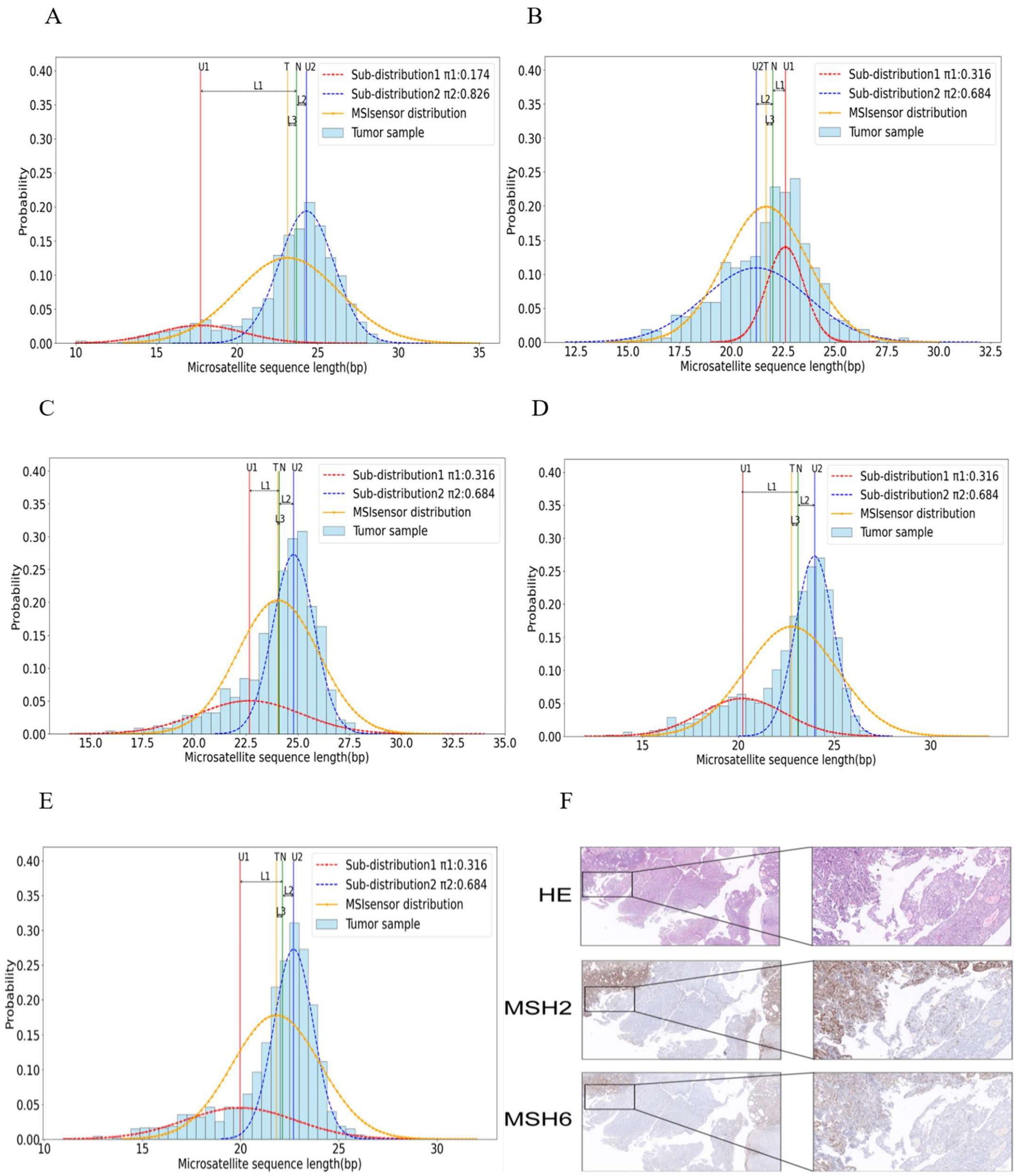

Figure L. IHC detection map and PCR microsatellite detection site length distribution map of case 14. **(A)** BAT26 microsatellite length distribution map **(B)** NR24 microsatellite length distribution map **(C)** BAT25 microsatellite length distribution map **(D)** NR27 microsatellite length distribution map **(E)** NR21 microsatellite length distribution map. **(F)** The figures of clonal immunohistochemical loss of MMR expression in tumor cells: clonal loss of MSH2 and MSH6 protein in the majority of tumor cells.

Table U. Classification of clonal microsatellite status for case 15.

Clone proportion	Site	μ	σ	π	Normal	MS	MSIsensor	MSIsensor-pro	MSINGS
[0.071, 0.068, 0.088, 0.773]	BAT26	24.45<br>20.62	1.74<br>2.57	0.773<br>0.227	23.58<br>2.42	MSI<br>MSI	MSS	MSS	MSS
	NR24	22.22<br>17.91	1.40<br>1.74	0.861<br>0.139	21.96<br>1.81	MSI<br>MSI	MSI	MSI	MSS
	BAT25	24.47<br>20.67	1.25<br>2.06	0.773<br>0.227	24.16<br>2.07	MSS<br>MSI	MSS	MSS	MSS
	NR27	19.12<br>23.68	1.93<br>1.36	0.227<br>0.773	23.25<br>2.04	MSI<br>MSI	MSI	MSI	MSS
	NR21	21.73	2.34	1	22.16<br>1.79	MSI	MSI	MSI	MSS
	MS1	12.5<br>13.8	1.52<br>0.62	0.227<br>0.773	13.63<br>0.99	MSI<br>MSI	MSS	MSS	MSS
	MS4	12.88	0.79	1	12.95<br>0.76	MSI	MSS	MSS	MSS
	MS5	17.44	1.16	1	17.61<br>1.10	MSI	MSS	MSS	MSS
	MS8	15.66	1.04	1	15.80<br>1.05	MSI	MSI	MSS	MSS
	MS9	15.63	1.24	1	15.66<br>1.16	MSS	MSS	MSS	MSS
	MS10	14.54	1.20	1	14.7<br>1.04	MSI	MSS	MSS	MSS
	MS12	14.35	1.24	1	14.45<br>1.17	MSS	MSS	MSS	MSS
	MS15	18.05<br>18.63	1.12<br>1.15	0.773<br>0.227	18.17<br>0.98	MSS<br>MSI	MSS	MSS	MSS
	MS22	15.33<br>16.56	1.87<br>1.42	0.227<br>0.773	16.48<br>1.49	MSI<br>MSS	MSS	MSS	MSS
	MS23	18.10	2.08	1	18.62<br>1.50	MSI	MSS	MSS	MSS
Sample							MSI	MSS	MSS
MANTIS								MSS	
IHC								partial MSI	

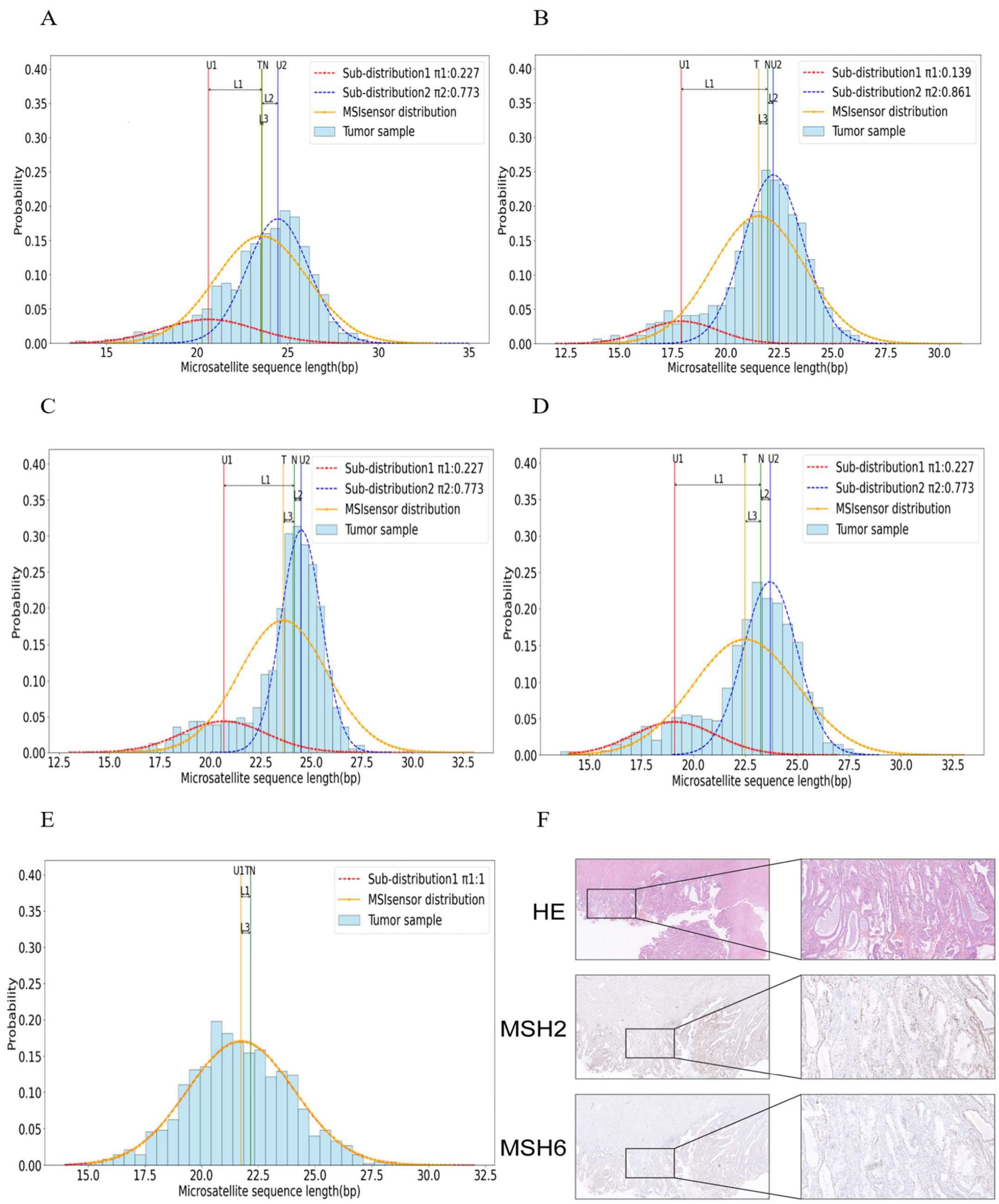

Figure M. IHC detection map and PCR microsatellite detection site length distribution map of case 15. **(A)** BAT26 microsatellite length distribution map **(B)** NR24 microsatellite length distribution map **(C)** BAT25 microsatellite length distribution map **(D)** NR27 microsatellite length distribution map **(E)** NR21 microsatellite length distribution map. **(F)** The figures of clonal immunohistochemical loss of MMR expression in tumor cells: clonal loss of MSH2 and MSH6 protein in focal tumor cells.

Table V. Classification of clonal microsatellite status for case 16.

Clone proportion	Site	μ	σ	π	Normal	MS	MSIsensor	MSIsensor-pro	MSINGS
[0.715, 0.19, 0.095]	BAT26	21.61	3.04	1	23.71<br>2.28	MSI	MSI	MSI	MSS
	NR24	21.83<br>18.97	1.69<br>2.43	0.81<br>0.19	21.94<br>1.83	MSS<br>MSI	MSI	MSI	MSS
	BAT25	21.12<br>23.40	2.37<br>1.43	0.285<br>0.715	23.49<br>1.97	MSI<br>MSS	MSI	MSI	MSS
	NR27	24.13<br>19.41<br>20.91	1.19<br>2.47<br>2.34	0.095<br>0.19<br>0.715	23.20<br>2.02	MSI<br>MSI<br>MSI	MSI	MSI	MSI
	NR21	21.43<br>22.41	1.99<br>0.82	0.715<br>0.285	21.70<br>1.68	MSI<br>MSI	MSS	MSS	MSS
	MS1	13.51<br>11.57	0.92<br>1.05	0.905<br>0.095	13.61<br>1.04	MSS<br>MSI	MSI	MSI	MSS
	MS4	12.89	0.73	1	12.93<br>0.71	MSS	MSS	MSS	MSS
	MS5	17.46	1.25	1	17.60<br>1.16	MSI	MSS	MSS	MSS
	MS8	16.36<br>15.09	0.89<br>1.29	0.81<br>0.19	16.34<br>1.08	MSS<br>MSI	MSI	MSI	MSS
	MS9	13.77<br>15.56<br>17.04	1.07<br>0.63<br>0.77	0.19<br>0.715<br>0.095	15.70<br>1.10	MSI<br>MSS<br>MSI	MSI	MSI	MSS
	MS10	12.61<br>14.75<br>16.04	0.98<br>0.55<br>0.68	0.19<br>0.715<br>0.095	14.77<br>1.02	MSI<br>MSS<br>MSI	MSI	MSI	MSS
	MS12	14.85<br>14.03	0.84<br>1.49	0.285<br>0.715	14.85<br>1.31	MSS<br>MSI	MSI	MSI	MSS
	MS15	18.04<br>15.79	0.92<br>1.13	0.905<br>0.095	18.10<br>1.10	MSS<br>MSI	MSS	MSS	MSS
	MS22	16.18<br>14.21	1.15<br>1.54	0.905<br>0.095	16.14<br>1.17	MSS<br>MSI	MSS	MSI	MSS
	MS23	17.11	1.74	1	17.72<br>1.39	MSI	MSI	MSI	MSS
Sample							MSI	MSI	MSS
MANTIS								MSS	
IHC								partial MSI	

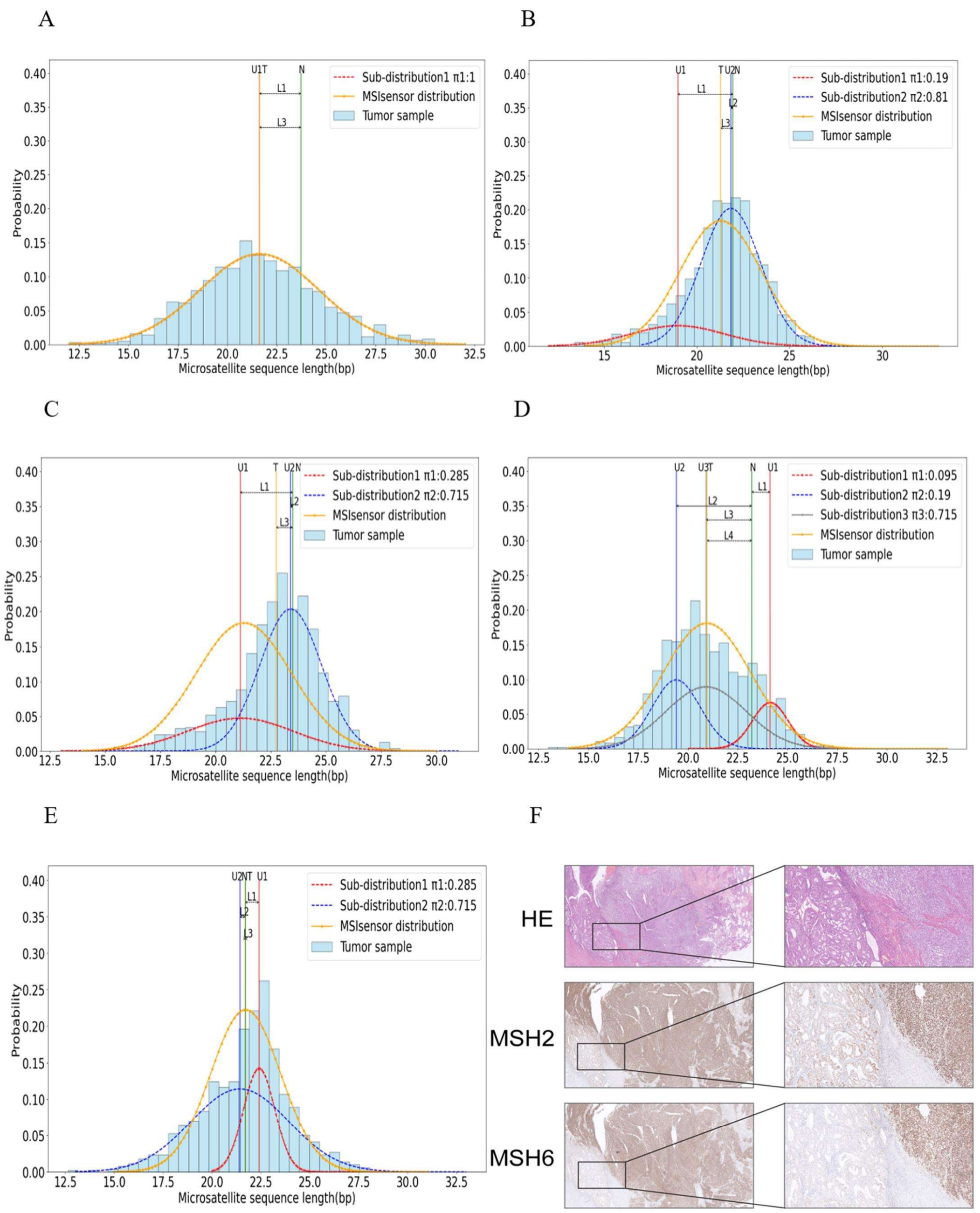

Figure N. IHC detection map and PCR microsatellite detection site length distribution map of case 16. **(A)** BAT26 microsatellite length distribution map **(B)** NR24 microsatellite length distribution map **(C)** BAT25 microsatellite length distribution map **(D)** NR27 microsatellite length distribution map **(E)** NR21 microsatellite length distribution map. **(F)** The figures of clonal immunohistochemical loss of MMR expression in tumor cells: clonal loss of MSH2 and MSH6 protein in focal tumor cells.
